# Intimate partner violence and depressive symptoms among ever-partnered Peruvian women: a repeated cross-sectional analysis of national surveys, 2015–2024

**DOI:** 10.3389/fpsyg.2026.1939992

**Published:** 2026-10-14

**Authors:** Miguel A. Arce-Huamani, Luked R. Picon-Flores, Fiorella F. Fuentes-Cosme, Maricarmen X. Benites-Pereda, Dyego Leandro Bezerra de Souza, Gabriel De la Cruz-Ku, Maritza M. Ortiz-Arica, J. Smith Torres-Roman, Luis M. Tasayco-Marquez

**Affiliations:** 1 Research for Evidence Care, and Public Health, Programa Academico de Medicina Humana, Facultad de Ciencias de la Salud, Universidad Privada Norbert Wiener, Lima, Peru; 2 Graduate Program in Public Health, Federal University of Rio Grande do Norte (UFRN), Natal, Brazil; 3 Universidad Cientifica del Sur, Lima, Peru; 4 Direccion de Politicas de Niñas, Niños y Adolescentes, Direccion General de Niñas, Niños y Adolescentes, Ministerio de la Mujer y Poblaciones Vulnerables, Lima, Peru; 5 Escuela de Posgrado, Universidad Continental, Lima, Peru; 6 Escuela de Posgrado, Universidad Tecnologica del Peru, Lima, Peru

**Keywords:** depressive symptoms, intimate partner violence, PHQ-9, SDG 3: Good health and well-being, women

## Abstract

**Background:**

Intimate partner violence (IPV) is a major public health concern associated with adverse mental health outcomes in women. We examined trends in depressive symptoms and temporal variation in their association with IPV among ever-partnered Peruvian women from 2015 to 2024.

**Methods:**

We conducted a repeated cross-sectional analysis using data from 10 consecutive national ENDES surveys. The primary weighted analytic sample included 101,145 women aged 15–49 years. Depressive symptoms were defined as a PHQ-9 score ≥10. Survey-weighted prevalence ratios were estimated using modified Poisson regression with robust variance.

**Results:**

Overall, 35.3% of women reported any IPV, including physical (28.0%), emotional (24.5%), and sexual IPV (6.1%). The weighted prevalence of depressive symptoms was 7.1%. The estimated annual relative change was +2.31% (95% CI: 1.03–3.61%; *p* < 0.001), with a non-monotonic annual pattern. Any IPV was associated with higher prevalence of depressive symptoms (aPR: 2.37; 95% CI: 2.18–2.58). Emotional, physical, and sexual IPV were also associated with depressive symptoms (aPRs: 2.32 [95% CI: 2.14–2.53], 2.21 [95% CI: 2.04–2.39], and 1.97 [95% CI: 1.80–2.17], respectively). The association varied across periods: 2015–2019, aPR 2.72; 2020–2021, 1.77; and 2022–2024, 2.29 (p for interaction <0.001).

**Conclusion:**

IPV was consistently associated with depressive symptoms, although the magnitude varied over time. These findings support integrated, confidential identification of IPV alongside timely mental health assessment and referral, without establishing the effectiveness of any specific screening strategy.

## 1 Introduction

Intimate partner violence (IPV) is a major public health concern closely linked to women’s mental health, and a meta-analysis of 201 studies involving 250,599 women found that IPV exposure was associated with approximately two- to threefold higher odds of depression (White et al., 2024). A systematic review of 11 longitudinal studies comprising 118,544 women further showed that IPV predicted subsequent depression, with a pooled odds ratio of 1.92 (95% CI: 1.28–2.86; Watson and Bitsika, 2025). Although the global age-standardized burden of major depressive disorder attributable to IPV declined between 1990 and 2019, inequalities between countries widened and vulnerability differed across women’s life stages (Guo et al., 2024). Mental health outcomes also vary by exposure characteristics, because greater IPV severity particularly psychological violence severity has been consistently associated with poorer depressive outcomes (White et al., 2024). In low- and middle-income countries, limited education, early marriage, economic disadvantage, rural residence, and violence-accepting gender norms shape IPV and its consequences (Gunarathne et al., 2023). Thus, the IPV–depression relationship is substantial, heterogeneous, and socially patterned.

In Peru, an analysis of 62,870 women from the 2015–2017 Demographic and Family Health Surveys estimated an overall IPV prevalence of 38.7%; psychological, physical, and sexual violence affected 26.8%, 31.2%, and 6.9%, respectively (Burgos-Muñoz et al., 2021). Another national analysis of 24,099 women surveyed during 2014–2016 reported prevalences of 6.98% for depressive symptoms and 64.72% for IPV, with exposed women having a 2.47-fold higher prevalence of depressive symptoms (Silva-Burga et al., 2022). Using ENDES 2019, low household autonomy was associated with a 15% higher IPV prevalence, and 40.1% of married or cohabiting women reported partner violence (Barón-Lozada et al., 2022); additionally, 42.0% had witnessed violence against their mother during childhood, which was associated with a 69% higher prevalence of recent IPV in adulthood (Bendezu-Quispe et al., 2024). Among pregnant women, ENDES 2013–2019 data showed that IPV was associated with more than twice the prevalence of depressive symptoms (aPR: 2.26; 95% CI: 1.88–2.73; Benites-Meza et al., 2023), whereas a pandemic-period study found depression in 62.17% and spousal violence in 17.6% of participants (Medina-Quispe et al., 2022). These findings indicate that IPV and depressive symptoms coexist across women’s life stages in Peru, although estimates vary by population and period.

Recent Peruvian evidence suggests important heterogeneity: IPV typologies combining form and severity showed progressively stronger associations with recent depression (Hernández and Ortega, 2025), while an ENDES 2023 analysis found adjusted prevalence ratios ranging from 1.37 to 2.71 across household wealth quintiles (Chaquila et al., 2025). Among 5,903 postpartum women surveyed during 2021–2023, depressive symptoms affected 6.32%, IPV affected 19.23%, and exposed women had 4.53 times the adjusted odds of depressive symptoms (Muñoz-Callupe et al., 2025). Measurement also matters in Peru’s multilingual setting: the Collao Quechua adaptation of the Woman Abuse Screening Tool showed high reliability and a correlation of 0.618 with PHQ-9 scores among 521 bilingual women (Calsina-Rosa et al., 2025). Studies from Zimbabwe and Colombia indicate that socioeconomic hardship, cumulative trauma, self-esteem, and social support shape the mental health burden associated with IPV (Machisa and Shamu, 2022; Molina and Matud, 2024), while longitudinal research suggests that greater violence severity predicts less favorable depression trajectories after separation (Ford-Gilboe et al., 2023).

Previous nationally representative studies have already demonstrated an association between IPV and depressive symptoms among Peruvian women, including analyses of ENDES 2014–2016 and subsequent survey periods (Silva-Burga et al., 2022; Benites-Meza et al., 2023; Chaquila et al., 2025). The contribution of the present study is therefore not to establish this association for the first time, but to provide an updated decade-long assessment using 10 consecutive national surveys, examine whether the magnitude of the association varied across distinct social periods, and describe the national temporal pattern of depressive symptoms over 2015–2024.

Temporal variation in the IPV–depressive symptoms association was considered plausible on substantive grounds. During 2020–2021, pandemic-related restrictions and changes in household conditions may have altered privacy during interviews, access to social and health services, help-seeking, and reporting of partner violence. At the same time, broader pandemic-related psychological distress among women without reported IPV could have reduced the relative contrast in depressive symptoms between IPV-exposed and unexposed women. After 2021, reopening, restoration of services, and changes in social interaction and survey implementation could again modify both reporting conditions and the observed association. On this conceptual basis, the study distinguished 2015–2019 as the pre-pandemic period, 2020–2021 as the pandemic-disruption period, and 2022–2024 as the post-acute pandemic period. These periods were defined to evaluate temporal heterogeneity rather than to imply that the pandemic caused changes in the IPV–depressive symptoms association.

The covariate strategy was similarly guided by a conceptual hierarchy rather than by statistical significance alone. Sociodemographic, socioeconomic, household, and geographic characteristics were treated as the primary adjustment set because they may be related to both a history of IPV and depressive symptoms. Partner alcohol consumption and intergenerational violence were considered additional contextual factors. In contrast, partner controlling behaviors were treated as conceptually proximal to emotional IPV because they may represent a component, correlate, or intermediate variable rather than a conventional confounder. Moreover, union status, employment, and number of children may themselves be influenced by previous IPV. Consequently, the extended adjustment model was not regarded as causally superior, and all adjusted estimates were interpreted as associations rather than causal effects.

Despite the existing evidence, national survey evidence covering a full decade remains limited regarding trends in depressive symptoms and temporal changes in the magnitude of their association with IPV among ever-partnered Peruvian women. A decade-long assessment can provide an updated population-based benchmark, identify periods in which the strength of the association differs, and clarify whether emotional, physical, and sexual IPV show comparable associations with depressive symptoms when examined separately. Therefore, this study aimed to examine national trends in depressive symptoms, assess their association with intimate partner violence, and evaluate temporal variation in the magnitude of this association among ever-partnered Peruvian women from 2015 to 2024.

## 2 Materials and methods

### 2.1 Study design and setting

We conducted a repeated cross-sectional study using secondary microdata from the Peruvian Demographic and Family Health Survey (ENDES), collected annually between 2015 and 2024 by the National Institute of Statistics and Informatics of Peru (INEI). Each ENDES round constitutes an independent, nationally representative survey; therefore, all time-trend estimates reflect population-level changes across calendar years rather than within-person longitudinal changes. No participant-level longitudinal follow-up was performed or assumed; the pooled dataset therefore represents independent annual cross-sectional samples rather than repeated observations of the same women.

ENDES uses a probabilistic, stratified, multistage, and clustered sampling design, with inferential domains that include the national, urban/rural, natural-region, and departmental levels, as specified in the official INEI technical documentation. The survey collects information on household characteristics, women’s sociodemographic profile, reproductive history, health conditions, and intimate partner violence. Data are collected through direct interviews conducted by trained fieldworkers using standardized questionnaires and official INEI field procedures (Instituto Nacional de Estadística e Informática, 2026).

The unit of analysis was the woman. We pooled harmonized annual ENDES files from 2015 through 2024 and applied the complex survey design in all descriptive and inferential analyses. Sampling weights, strata, and clustered primary sampling units were incorporated to account for the ENDES complex survey design and to support population-based estimation within the health-module sampling framework. The study was reported according to the Strengthening the Reporting of Observational Studies in Epidemiology (STROBE) guidelines.

### 2.2 Population and sample

The target population comprised Peruvian women aged 15–49 years who had ever been married, had ever cohabited with a partner, or were formerly in such a union and who were eligible for the intimate partner violence module. For the purposes of this study, “ever-partnered” was operationalized as having ever been married, having ever cohabited, or being formerly in a marital or cohabiting union; women whose only relationship history consisted of non-cohabiting dating relationships were not included in this analytic definition. The analytic population was restricted to ever-partnered women with complete PHQ-9 information and sufficient valid intimate partner violence data.

Women were included if they met all of the following criteria: (1) age 15–49 years at the time of interview; (2) history of marriage, cohabitation, or union; (3) complete information for the nine PHQ-9 items; and (4) valid information on intimate partner violence by the current or most recent husband/partner. Women were excluded if they were never partnered, lacked PHQ-9 information, lacked intimate partner violence information, or could not be linked across the required ENDES modules using individual and household identifiers.

Across the 10 annual women’s files, 372,972 records were available before module-specific selection and linkage. A total of 136,427 women had records available in the linked health and domestic-violence module intersection. This reduction reflects the module-specific sampling and eligibility procedures of ENDES, together with the requirement that records be jointly available across the modules used in the analysis, and should not be interpreted solely as missing data or unsuccessful linkage. Among these women, 111,912 were ever-partnered. Three ever-partnered women had incomplete PHQ-9 information, and 10,533 additional women lacked sufficient valid IPV information, resulting in 101,376 women who met the substantive analytic criteria. Of these, 101,145 had a positive, non-missing health-module sampling weight and constituted the primary weighted analytic sample; 197 had a missing health-module weight and 34 had a zero weight. Year-specific sample-selection counts are provided in Supplementary Table S1. For temporal analyses, survey years were grouped into three analytical periods: 2015–2019, 2020–2021, and 2022–2024. These periods were defined to distinguish the pre-pandemic baseline, the period of pandemic-related survey and social disruption, and the post-2021 period.

### 2.3 Data collection

ENDES data were collected by trained interviewers using standardized questionnaires under INEI supervision. The present study used information from the women’s individual file, domestic violence module, health module, household characteristics file, household members file, and complementary women’s modules. These files were linked using harmonized household, cluster, and woman-level identifiers.

Depressive symptoms were obtained from the ENDES health module, which includes the nine items of the Patient Health Questionnaire (PHQ-9). Intimate partner violence variables were obtained from the domestic-violence component of the ENDES individual women’s questionnaire, as documented in the official survey materials (Instituto Nacional de Estadística e Informática, 2026). The violence module records acts committed by the current or most recent husband/partner. Accordingly, IPV was interpreted as history of partner violence rather than incident IPV.

Administration of the ENDES domestic-violence section was conditional on both selection of the woman for that section and the availability of complete privacy. Official ENDES procedures instruct interviewers not to continue with the domestic-violence questions when another person is present and complete privacy cannot be obtained (Instituto Nacional de Estadística e Informática, 2024). Accordingly, women without sufficient valid IPV information were not classified as unexposed; they were excluded when the analytic IPV sample was defined. The harmonized analytic files used in this study did not retain a consistently comparable indicator across all 10 survey rounds that allowed privacy-related non-administration to be distinguished from other causes of incomplete IPV information. Therefore, the number of women excluded specifically because privacy could not be ensured could not be quantified separately.

Sociodemographic, territorial, and household-level covariates were obtained from the women’s individual file and household files. These included age, educational level, union status, current work status, number of children living in the household, mother tongue, area of residence, natural region, wealth quintile, partner alcohol consumption, partner controlling behaviors, and whether the woman’s father had beaten her mother.

### 2.4 Variables

The primary outcome was depressive symptoms, assessed using the nine-item Patient Health Questionnaire (PHQ-9), which evaluates depressive symptom frequency during the preceding 2 weeks (Kroenke et al., 2001). In ENDES, the nine original health-module variables were QS700A–QS700I: little interest or pleasure (QS700A), depressed mood (QS700B), sleep problems (QS700C), tiredness or low energy (QS700D), poor appetite or overeating (QS700E), difficulty concentrating (QS700F), psychomotor slowing or restlessness (QS700G), thoughts of self-harm or death (QS700H), and negative feelings about oneself (QS700I). Each item was coded from 0 to 3 according to symptom frequency: 0 = not at all, 1 = several days (1–6 days), 2 = most days (7–11 days), and 3 = nearly every day (12 or more days). The nine responses were summed to obtain a total score ranging from 0 to 27. All nine items were required to have valid responses; if any PHQ-9 item was missing or invalid, the total score was not calculated and the woman was considered to have incomplete PHQ-9 information for the analytic sample.

A PHQ-9 total score ≥10 was used to identify clinically relevant depressive symptoms, corresponding to at least moderate symptom severity and a commonly used screening threshold supported by the original validation study (Kroenke et al., 2001). Women with scores <10 were classified as not having depressive symptoms. The PHQ-9 has also been psychometrically evaluated in Peru using nationally representative ENDES data. Villarreal-Zegarra et al. (2019), using data from 30,449 Peruvian adults, supported a unidimensional structure, measurement invariance across major demographic groups, and high internal consistency (Cronbach’s α = 0.87; McDonald’s ω = 0.87). More recent evidence in Peruvian adolescents has also supported its unidimensional structure, measurement invariance by sex, and adequate reliability (Navarro-Loli et al., 2026). Review of the annual ENDES health-module dictionaries confirmed that QS700A–QS700I, the 14-day reference period, and the 0–3 response structure were retained throughout 2015–2024. The PHQ-9 was therefore used as a screening measure of depressive symptoms and not as a diagnostic instrument for major depressive disorder. Operational definitions, source variables, and recoding rules for all study variables are provided in Supplementary Table S2.

The main exposure was intimate partner violence perpetrated by the current or most recent husband/partner. The IPV measures represented ever-reported experience of the specified acts from that partner and therefore did not represent incident IPV or exclusively violence occurring during the previous 12 months. Although ENDES subsequently asks about the frequency of some reported acts during the preceding 12 months, those frequency responses were not used to define the exposures in the present analysis. Emotional IPV was defined using the original ENDES summary variable D104 (“ever any emotional violence”), coded 1 for yes and 0 for no. The associated emotional-violence block includes D103A (humiliation in front of others), D103B (threatened harm to the woman or someone close to her), D103C (insulted her or made her feel bad), and D103D (threatened to leave, take the children, or withdraw economic support), together with country-specific emotional-abuse fields where present. D104 itself was used directly and was not reconstructed from the individual D103 items.

Physical IPV was defined as the presence of either less-severe or severe physical violence. D106 is the original ENDES binary summary variable for less-severe physical violence based on D105A–D105D: pushing, shaking, or throwing something at the woman; slapping her or, from 2019 onward, slapping or twisting her arm; hitting her with a fist or an object that could cause harm; and kicking or dragging her. D107 is the original ENDES binary summary variable for severe physical violence based on D105E–D105G: attempted strangulation or burning, threatening with a knife, firearm, or other weapon, and attacking with such a weapon. Physical IPV was coded as present when D106 = 1 or D107 = 1.

Sexual IPV was defined using the original ENDES summary variable D108, based on D105H–D105I, which assess physical force to obtain unwanted sexual intercourse and forced sexual acts not approved by the woman; D108 = 1 was classified as sexual IPV. Any IPV was an author-derived composite coded 1 when emotional, physical, or sexual IPV was present and 0 only when all three subtypes were negative. Original missing or non-applicable values were never recoded as absence of violence; women without sufficient valid source information were classified as having incomplete IPV information.

During harmonization, the original ENDES variables D104, D106, D107, and D108 were copied into the standardized fields raw_d104, raw_d106, raw_d107, and raw_d108, respectively. Thus, the raw_ prefix denotes an author-assigned harmonized copy of an original ENDES field rather than a newly constructed violence measure. The binary coding of D104, D106, D107, and D108 was retained throughout 2015–2024. Review of the annual questionnaires and dictionaries identified generally stable content, although D105B changed from referring to slapping in earlier rounds to slapping or twisting the arm from 2019 onward. Because the official ENDES summary variables were used consistently across years, this difference was documented rather than manually recoded. Detailed source-item definitions and year-specific harmonization are presented in Supplementary Table S3.

The main time variable was survey year, analyzed annually from 2015 to 2024. For adjusted period analyses and interaction models, survey year was grouped into 2015–2019, 2020–2021, and 2022–2024. The three periods were not defined to have equal duration and were not part of a preregistered analysis plan. Rather, they were specified as substantively motivated calendar periods representing the pre-pandemic years (2015–2019), the period of greatest COVID-19-related disruption to survey implementation and social conditions (2020–2021), and the subsequent period (2022–2024). Official INEI documentation indicates that ENDES fieldwork changed substantially in 2020: telephone interviewing was introduced during the COVID-19 emergency, modified questionnaires were used during the interruption of regular fieldwork, and in-person interviewing was subsequently resumed progressively under biosafety procedures. In 2021, ENDES documentation continued to describe both direct in-person and telephone interviewing. These changes provide a substantive rationale for distinguishing 2020–2021, but the available harmonized microdata did not contain a consistently comparable respondent-level indicator of interview mode or interview privacy across all survey years. Consequently, the period classification was used to evaluate temporal heterogeneity and should not be interpreted as identifying a causal effect of the pandemic.

Covariate selection was guided by a conceptual causal-role framework rather than by bivariate statistical significance. Survey period, age, educational level, area of residence, natural region, household wealth, and mother tongue were treated as background sociodemographic or structural characteristics that could be related to both a history of IPV and depressive symptoms. Union status, current employment, and number of children living in the household were included in the primary sociodemographic adjustment model but were recognized as variables of uncertain temporal position because they could either precede IPV or be influenced by previous IPV. Partner alcohol consumption and intergenerational violence, represented by whether the woman’s father had beaten her mother, were considered additional contextual factors that could plausibly precede or contribute to both IPV and adverse mental health. Partner controlling behaviors were treated separately because they are conceptually proximal to emotional IPV and may represent a component, correlate, or intermediate variable rather than a conventional confounder. No covariate was included solely as a precision variable. Because the timing of IPV relative to several time-varying covariates could not be established in the repeated cross-sectional data, this framework was used to organize plausible covariate roles rather than to identify a formal causal effect; all adjusted estimates were therefore interpreted as associations.

Age was categorized as 15–19, 20–29, 30–39, and 40–49 years. Educational level was categorized as no formal/initial education, primary, secondary, and higher education. Union status was categorized as currently married/cohabiting and formerly in union. Current work status was categorized as no, yes, and missing/not reported. Number of children living in the household was categorized as 0, 1–2, and ≥3. Area of residence was categorized as urban and rural. Natural region was categorized as Coast, Highlands, Rainforest, and Metropolitan Lima. Wealth quintile was categorized as poorest, poorer, middle, richer, and richest. Mother tongue was categorized as Spanish/Castilian, Indigenous language, and other/not reported. Partner alcohol consumption, partner controlling behaviors, and father beating mother were categorized as no, yes, and missing/not reported when applicable.

Missing data were handled according to the analytical role of each variable. Complete information for all nine PHQ-9 items and sufficient valid IPV information were required for inclusion in the substantive analytic sample; missing outcome or exposure information was therefore not imputed. Within the primary weighted analytic sample, missing/not-reported categories were retained for current work status, partner alcohol consumption, partner controlling behaviors, and father beating mother. Overall, 8,110 of 101,145 women (8.0%) had at least one missing value across these four covariates. Missingness was not evenly distributed across survey years: the proportion with at least one missing covariate was 47.1% in 2020 and 10.8% in 2021, compared with approximately 2.9–3.8% in the remaining survey years. This concentration was driven primarily by current work status and partner alcohol consumption, particularly in 2020. Year-specific missingness patterns and a descriptive comparison of women included in and excluded from the analytic sample are provided in Supplementary Table S1.

The primary models retained missing/not-reported categories to preserve the analytic sample, but coefficients for these categories were treated as nuisance parameters and were not interpreted as epidemiological associations. To evaluate the robustness of the principal IPV estimates to this approach, a complete-case sensitivity analysis was conducted among the 93,035 women with observed information for all Model 2 covariates. Multiple imputation was not performed because missingness was strongly concentrated in specific survey years and the missing-at-random assumption required for an imputation model could not be confidently justified across the pooled surveys. The complete-case estimates were therefore used as the principal sensitivity analysis for the handling of missing covariate information.

### 2.5 Data analysis

All analyses accounted for the complex sampling design of ENDES. Because depressive symptoms measured with the PHQ-9 were obtained from the health module administered to one selected household member aged ≥15 years, the primary analyses used the corresponding health-module sampling weight. Specifically, PESO15_AJUS/1,000,000 was used for the 2015 survey and PESO15_AMAS/1,000,000 for the 2016–2024 surveys. This weight is specific to the health-module selection and should not be interpreted as a domestic-violence-specific weight. The domestic-violence module involves a separate within-household selection process; however, a distinct domestic-violence sampling weight was not consistently available across all 10 survey rounds, and ENDES does not provide an official joint sampling weight for the intersection of the health and domestic-violence subsamples. Therefore, no composite weight was constructed by multiplying the health-, women’s-, or violence-module weights. Observations with a missing or zero health-module sampling weight were excluded from survey estimation.

For pooled 2015–2024 analyses, the annual health-module weights were retained on their original annual scale after division by 1,000,000; no additional cross-year rescaling was applied. Primary sampling unit and stratum identifiers were concatenated with survey year so that identifiers from different annual survey rounds were treated as distinct sampling units. To preserve the survey design when estimating results for the analytic population, all 136,021 women with a positive health-module weight in the linked health and domestic-violence module intersection were retained in the survey-design object, and the 101,145 women meeting the substantive analytic criteria were specified as the survey subpopulation rather than deleting non-analytic observations before variance estimation. Reanalysis using this domain-estimation approach produced the same principal point estimates and materially unchanged standard errors and confidence intervals. Accordingly, estimates from the linked analytical subsample should be interpreted as survey-weighted, population-based estimates derived from national ENDES surveys rather than as estimates demonstrated to retain the full national representativeness of the original ENDES design.

Descriptive analyses were performed to characterize the study population. Absolute frequencies were reported as unweighted counts, while percentages were estimated using the ENDES health-module sampling weight described above. The prevalence of depressive symptoms was estimated overall, annually, and according to history of IPV.

Annual weighted prevalence estimates of depressive symptoms were calculated for each survey year and displayed graphically for the total analytic population and stratified by history of any IPV. The temporal trend was estimated at the individual-record level using the pooled 2015–2024 survey data rather than by fitting a model to the 10 annual prevalence estimates. Specifically, an unadjusted survey-weighted generalized linear model with a Poisson family and log link was fitted with depressive symptoms (PHQ-9 ≥ 10) as the binary outcome and survey year as a continuous predictor. The model incorporated the ENDES health-module sampling weight, year-specific primary sampling units and strata, and the analytic subpopulation described above; variances were estimated using the design-based Taylor linearization implemented for complex survey data. The coefficient for survey year (βyear) was exponentiated and transformed as 100 × [exp(βyear) − 1] to obtain the estimated annual relative change in the prevalence of depressive symptoms, with its corresponding 95% confidence interval. Thus, the resulting percentage represents a relative annual change in prevalence and not an annual percentage-point difference. Because Joinpoint regression was not used, this quantity was reported as annual relative change rather than average annual percent change.

As a sensitivity analysis, survey year was also treated as a categorical variable rather than as a continuous variable. The 10 survey years (2015–2024) were entered separately in the model, with 2015 as the reference year. This analysis was performed to evaluate whether the temporal pattern of depressive symptoms remained evident without assuming a constant log-linear change across years. A design-adjusted Wald test was used to assess overall differences across survey years.

Bivariate associations between depressive symptoms and each exposure or covariate were evaluated using survey-weighted prevalence estimates and crude prevalence ratios. Crude prevalence ratios and 95% confidence intervals were estimated using modified Poisson regression with a log link and robust variance, accounting for the complex survey design.

Multivariable analyses were performed using modified Poisson regression with robust variance to estimate adjusted prevalence ratios and 95% confidence intervals. This modeling strategy was selected because depressive symptoms were not treated as a rare outcome and odds ratios from logistic regression could overestimate the association. Covariates were retained based on conceptual relevance rather than bivariate statistical significance.

Two prespecified adjusted models were fitted for each IPV measure. Model 1 was considered the primary adjustment model and included survey period, age group, educational level, union status, current work status, number of children living in the household, area of residence, natural region, wealth quintile, and mother tongue. Model 1 was intended as the primary sociodemographic adjustment model rather than as a formally identified minimal sufficient causal adjustment set. Model 2 was treated as an extended contextual model and additionally included partner alcohol consumption, partner controlling behaviors, and father beating mother. Model 2 was not interpreted as a causally superior or “fully adjusted” model because controlling behaviors are conceptually close to emotional IPV and may represent a component, correlate, or intermediate variable, while the temporal ordering of several partner- and household-related variables cannot be established in a repeated cross-sectional design.

To assess potential overadjustment related specifically to partner controlling behaviors, we conducted a sensitivity analysis that retained all Model 1 covariates together with partner alcohol consumption and father beating mother but excluded partner controlling behaviors. This sensitivity model was fitted separately for any IPV, emotional IPV, physical IPV, and sexual IPV. Agreement in the direction and magnitude of the estimates across Model 1, the sensitivity model, and Model 2 was used to assess the robustness of the main associations to the alternative adjustment specification.

Separate adjusted models were fitted for any IPV, emotional IPV, physical IPV, and sexual IPV. IPV types were not entered simultaneously in the same model to avoid overadjustment and instability due to conceptual and statistical overlap between types of violence. Accordingly, subtype-specific estimates were not interpreted as mutually independent effects.

To assess temporal heterogeneity in the IPV–depressive symptoms association, we evaluated an interaction between any IPV and survey period. Period-specific crude and adjusted prevalence ratios were estimated for the association between any IPV and depressive symptoms. The global interaction *p*-value was obtained using a design-adjusted Wald test. Adjusted models for the interaction included age group, educational level, union status, current work status, number of children living in the household, area of residence, natural region, wealth quintile, mother tongue, partner alcohol consumption, partner controlling behaviors, and father beating mother. To assess whether the temporal interaction was sensitive to pandemic-period covariate missingness and the extended adjustment set, we repeated the IPV × survey period interaction using Model 1 covariates only. We also performed a complete-case interaction analysis using Model 2 among women with observed information for all Model 2 covariates. For both sensitivity analyses, period-specific adjusted prevalence ratios and the global design-adjusted Wald test for the interaction were compared with the primary Model 2 results.

Multicollinearity was evaluated using generalized variance inflation factors (GVIFs) for terms included in Model 2. Because several categorical predictors had more than one degree of freedom, the reported diagnostic was the degrees-of-freedom-adjusted quantity GVIF^(1/[2 × df]), which facilitates comparison across model terms of different dimensionality. These diagnostics were used solely to evaluate numerical collinearity and were not used for covariate selection or interpreted as evidence against conceptual overadjustment. The corresponding values are reported in Supplementary Table S4. Year-specific weighted prevalence estimates of depressive symptoms were also calculated overall and by history of IPV.

All prevalence ratios and adjusted prevalence ratios were reported with two decimal places. *p*-values were reported as exact values when appropriate and as <0.001 for very small values. Statistical significance was defined using a two-sided *p*-value <0.05.

## 3 Results

### 3.1 Characteristics of the study population

After harmonization of the 2015–2024 ENDES files, 101,376 ever-partnered women aged 15–49 years met the substantive inclusion criteria. Of these, 101,145 had a positive, non-missing health-module sampling weight and constituted the primary weighted analytic sample. Absolute frequencies were unweighted, whereas percentages were estimated using the ENDES health-module sampling weight. Of the weighted sample, 50.0% were surveyed during 2015–2019, 19.5% during 2020–2021, and 30.5% during 2022–2024. Women aged 30–39 years accounted for the largest proportion (38.0%), followed by those aged 20–29 years (31.6%) and 40–49 years (27.0%). Most participants had completed secondary education (45.6%), were currently married or cohabiting (81.8%), were working at the time of the survey (58.9%), and lived in urban areas (77.4%). In addition, 71.7% had one or two children living in the household, and 67.2% reported Spanish or Castilian as their mother tongue. Among ever-partnered women with a positive health-module sampling weight in the linked health and domestic-violence module intersection, those excluded because of incomplete PHQ-9 or IPV information were more frequently aged 40–49 years, formerly in union, and urban residents than women included in the primary analytic sample; detailed descriptive comparisons are provided in Supplementary Table S1.

Overall, 35.3% of women reported a history of any intimate partner violence. Physical IPV was reported by 28.0%, emotional IPV by 24.5%, and sexual IPV by 6.1%. Half of the weighted sample reported controlling behaviors by their partner (50.0%), 75.8% reported that their partner consumed alcohol, and 41.0% indicated that their father had physically assaulted their mother. The weighted prevalence of depressive symptoms was 7.1% (Table 1).

**Table 1:** Characteristics of the study population, Peru, ENDES 2015–2024.

General characteristics	*N* = 101,145	%
Total women	101,145	100.0
Survey period		
2015–2019	51,366	50.0
2020–2021	19,190	19.5
2022–2024	30,589	30.5
Age group		
15–19 years	2,662	3.3
20–29 years	35,579	31.6
30–39 years	43,072	38.0
40–49 years	19,832	27.0
Educational level		
No formal/initial	1,870	1.7
Primary	21,893	18.6
Secondary	46,923	45.6
Higher	30,459	34.2
Union status		
Currently married/cohabiting	83,549	81.8
Formerly in union	17,596	18.2
Currently working		
No	36,327	36.2
Yes	60,138	58.9
Missing/not reported	4,680	4.9
Number of children living in household		
0	7,967	9.7
1–2	71,260	71.7
≥3	21,918	18.7
Mother tongue		
Spanish/Castilian	61,250	67.2
Indigenous language	39,804	32.7
Other/not reported	91	0.1
Area of residence		
Urban	67,455	77.4
Rural	33,690	22.6
Natural region		
Coast	28,943	25.7
Highlands	35,469	26.8
Rainforest	26,282	14.8
Metropolitan Lima	10,451	32.8
Wealth quintile		
Poorest	30,571	20.9
Poorer	28,724	24.0
Middle	19,728	21.5
Richer	13,550	18.6
Richest	8,572	15.0
Any intimate partner violence		
No IPV	64,533	64.7
Any IPV	36,612	35.3
Emotional IPV		
No	75,999	75.5
Yes	25,146	24.5
Physical IPV		
No	71,713	72.0
Yes	29,432	28.0
Sexual IPV		
No	94,714	93.9
Yes	6,431	6.1
Partner drinks alcohol		
No	19,420	21.2
Yes	79,203	75.8
Missing/not reported	2,522	3.0
Controlling behaviors by partner		
No	50,828	50.0
Yes	50,287	50.0
Missing/not reported	30	0.0
Father beat mother		
No	55,014	55.8
Yes	42,525	41.0
Missing/not reported	3,606	3.2
Main outcome		
No depressive symptoms	94,374	92.9
Depressive symptoms	6,771	7.1

N values are unweighted; percentages are weighted using the ENDES health-module sampling weight for persons aged ≥15 years (PESO15_AJUS/1,000,000 in 2015 and PESO15_AMAS/1,000,000 in 2016–2024). The primary weighted analytic sample comprised 101,145 ever-partnered women aged 15–49 years with complete PHQ-9 information, sufficient valid intimate partner violence information, and a positive, non-missing health-module sampling weight. Percentages may not sum exactly to 100% because of rounding. Depressive symptoms were defined as a PHQ-9 score ≥10. IPV represents ever-reported partner violence perpetrated by the current or most recent husband/partner rather than incident IPV. Explicit missing/not-reported categories were retained for current work status, partner alcohol consumption, partner controlling behaviors, and father beating mother to preserve observations in the primary analysis; these categories should not be interpreted as substantive exposure groups. Source: ENDES 2015–2024.

### 3.2 Trends in depressive symptoms

The weighted prevalence of depressive symptoms showed a non-monotonic pattern across the study period. It declined from 7.5% in 2015 to 5.7% in 2020, subsequently increased to 8.4% in 2023, and was 8.2% in 2024. The log-linear model estimated an annual relative change of +2.31% between 2015 and 2024 (95% CI: 1.03–3.61%; *p* < 0.001). Across all survey years, depressive symptoms were consistently more prevalent among women with a history of any IPV than among those who did not report IPV (Figure 1). In the sensitivity analysis treating survey year as a categorical variable, overall differences across survey years remained statistically significant (design-adjusted Wald *p* < 0.001), confirming temporal heterogeneity without imposing a constant log-linear change across years. Year-specific weighted prevalence estimates overall and by history of IPV, together with the categorical-year sensitivity analysis, are presented in Supplementary Table S5.

**Figure 1:**
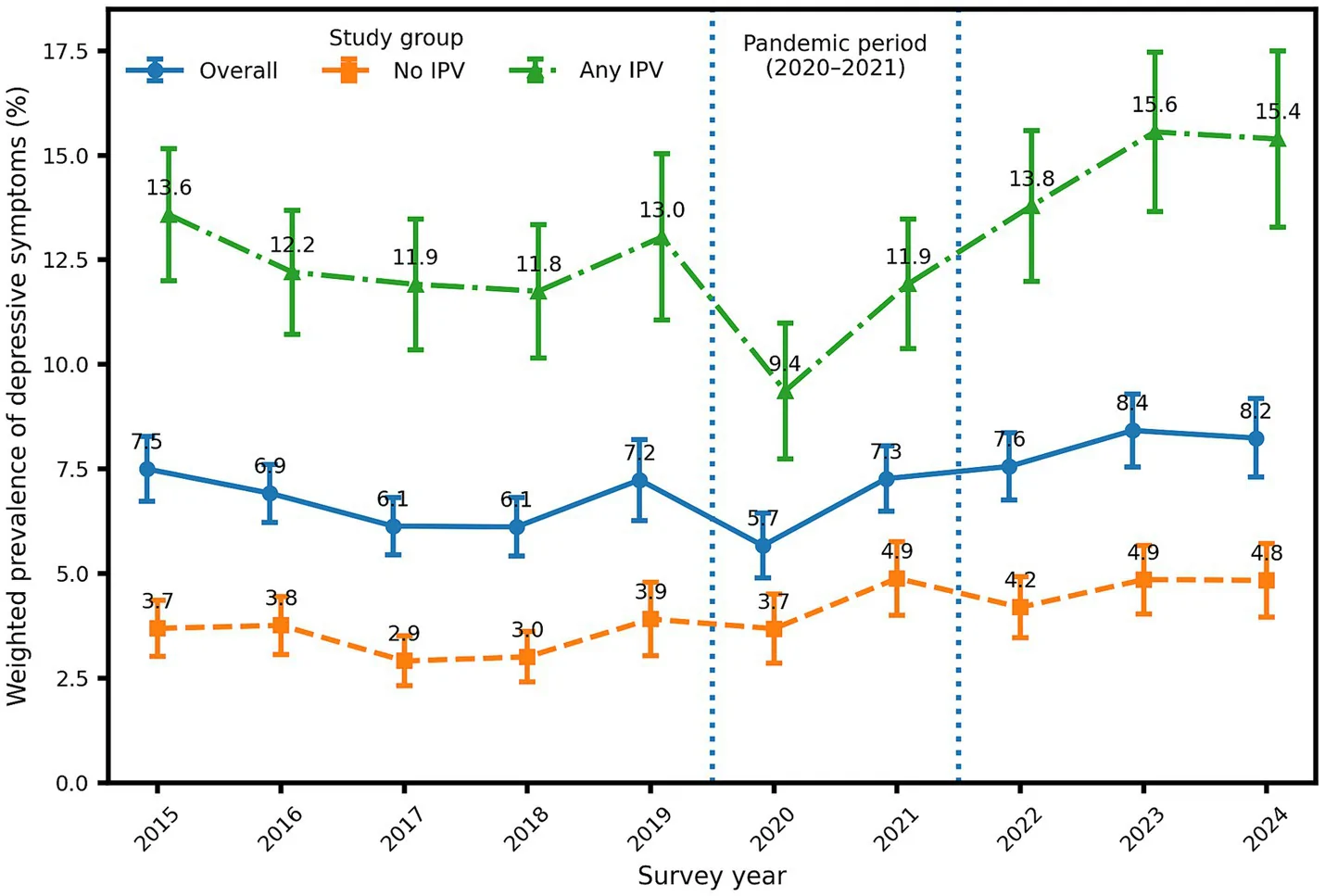
Annual weighted prevalence of depressive symptoms among ever-partnered Peruvian women, overall and according to intimate partner violence history, ENDES 2015–2024. Points represent year-specific weighted prevalence estimates and vertical error bars indicate 95% confidence intervals. Depressive symptoms were defined as a PHQ-9 score ≥10. Estimates were obtained using the ENDES health-module sampling weight and accounted for the complex survey design. The dotted vertical lines delimit the 2020–2021 pandemic period. Lines connect the observed annual estimates only and do not represent a smoothed or fitted trajectory. The estimated annual relative change for the overall analytic sample was +2.31% (95% CI: 1.03–3.61%; *p* < 0.001); this estimate summarizes the overall temporal tendency and should not be interpreted as a uniform year-to-year increase.

### 3.3 Bivariate associations with depressive symptoms

The weighted prevalence of depressive symptoms was 12.8% among women who reported any IPV, compared with 4.0% among those without a history of IPV. In the crude analysis, women who reported any IPV had 3.22 times the prevalence of depressive symptoms observed among women without IPV (PR = 3.22; 95% CI: 2.99–3.47; *p* < 0.001). Emotional IPV was associated with a 3.19-fold higher prevalence of depressive symptoms (PR = 3.19; 95% CI: 2.97–3.42; *p* < 0.001), while physical IPV was associated with a 3.03-fold higher prevalence (PR = 3.03; 95% CI: 2.82–3.25; *p* < 0.001). Women who reported sexual IPV had the highest prevalence of depressive symptoms, at 19.9%, corresponding to a crude PR of 3.17 (95% CI: 2.91–3.45; *p* < 0.001).

Several sociodemographic and partner-related characteristics were also associated with depressive symptoms in the crude analyses. Higher prevalences were observed among women aged 30–49 years, those formerly in union, women with lower educational attainment, those living in the Highlands, and those whose mother tongue was an Indigenous language. Depressive symptoms were also more prevalent among women whose partners consumed alcohol or displayed controlling behaviors and among women who reported that their father had physically assaulted their mother. In contrast, rural residence and having one or more children living in the household were associated with a lower crude prevalence of depressive symptoms (Table 2).

**Table 2:** Bivariate prevalence and crude association with depressive symptoms among ever-partnered Peruvian women, ENDES 2015–2024.

Variable	Weighted prevalence of depressive symptoms (%)	Crude PR (95% CI)	*p*-value
Any intimate partner violence			
No IPV	4.0	Reference	—
Any IPV	12.8	3.22 (2.99–3.47)	<0.001
Emotional IPV			
No	4.6	Reference	—
Yes	14.8	3.19 (2.97–3.42)	<0.001
Physical IPV			
No	4.5	Reference	—
Yes	13.8	3.03 (2.82–3.25)	<0.001
Sexual IPV			
No	6.3	Reference	—
Yes	19.9	3.17 (2.91–3.45)	<0.001
Survey period			
2015–2019	6.8	Reference	—
2020–2021	6.5	0.96 (0.87–1.06)	0.438
2022–2024	8.1	1.19 (1.10–1.29)	<0.001
Age group			
15–19 years	6.6	1.09 (0.85–1.39)	0.489
20–29 years	6.0	Reference	—
30–39 years	6.8	1.12 (1.03–1.22)	0.009
40–49 years	8.9	1.47 (1.34–1.62)	<0.001
Educational level			
No formal/initial	8.0	1.42 (1.14–1.75)	0.001
Primary	8.0	1.41 (1.27–1.55)	<0.001
Secondary	7.8	1.38 (1.26–1.50)	<0.001
Higher	5.7	Reference	—
Union status			
Currently married/cohabiting	6.0	Reference	—
Formerly in union	11.9	1.98 (1.83–2.13)	<0.001
Currently working			
No	6.3	Reference	—
Yes	7.7	1.23 (1.14–1.33)	<0.001
Missing/not reported	5.9	0.93 (0.77–1.13)	0.484
Number of children living in household			
0	10.0	Reference	—
1–2	6.6	0.66 (0.59–0.74)	<0.001
≥3	7.6	0.76 (0.67–0.86)	<0.001
Area of residence			
Urban	7.4	Reference	—
Rural	6.1	0.82 (0.76–0.88)	<0.001
Natural region			
Coast	6.7	Reference	—
Highlands	8.0	1.19 (1.10–1.29)	<0.001
Rainforest	5.6	0.84 (0.76–0.92)	<0.001
Metropolitan Lima	7.4	1.10 (0.99–1.22)	0.079
Wealth quintile			
Poorest	6.4	1.14 (0.99–1.32)	0.066
Poorer	7.8	1.39 (1.20–1.60)	<0.001
Middle	7.8	1.40 (1.21–1.63)	<0.001
Richer	7.5	1.35 (1.15–1.59)	<0.001
Richest	5.6	Reference	—
Mother tongue			
Spanish/Castilian	6.8	Reference	—
Indigenous language	7.8	1.15 (1.07–1.23)	<0.001
Other/not reported	6.9	1.01 (0.27–3.82)	0.984
Partner drinks alcohol			
No	6.3	Reference	—
Yes	7.4	1.18 (1.07–1.29)	<0.001
Missing/not reported	5.4	0.86 (0.66–1.13)	0.287
Controlling behaviors by partner			
No	4.2	Reference	—
Yes	10.0	2.39 (2.21–2.58)	<0.001
Missing/not reported	1.7	0.41 (0.06–3.08)	0.389
Father beat mother			
No	5.6	Reference	—
Yes	9.0	1.60 (1.49–1.72)	<0.001
Missing/not reported	8.9	1.59 (1.31–1.92)	<0.001

Percentages are weighted using the ENDES health-module sampling weight for persons aged ≥15 years (PESO15_AJUS/1,000,000 in 2015 and PESO15_AMAS/1,000,000 in 2016–2024). Crude prevalence ratios (PRs) and 95% confidence intervals (CIs) were estimated using survey-weighted modified Poisson regression with a log link and robust variance, accounting for sampling weights, strata, and clustered primary sampling units. Depressive symptoms were defined as a PHQ-9 score ≥10. IPV represents ever-reported partner violence perpetrated by the current or most recent husband/partner rather than incident IPV. Missing/not-reported categories were retained for current work status, partner alcohol consumption, partner controlling behaviors, and father beating mother; estimates for these categories are presented to document the analytical coding and should not be interpreted as substantive epidemiological associations. Reference categories are indicated by “Reference.” Source: ENDES 2015–2024.

### 3.4 Adjusted association between intimate partner violence and depressive symptoms

After adjustment for survey period, age group, educational level, union status, current work status, number of children living in the household, area of residence, natural region, wealth quintile, and mother tongue, all IPV measures remained significantly associated with depressive symptoms. In Model 1, the adjusted prevalence ratios ranged from 2.44 for sexual IPV to 2.86 for any IPV.

The magnitude of the associations was attenuated after further adjustment for partner alcohol consumption, controlling behaviors by the partner, and the history of the participant’s father physically assaulting her mother. Nevertheless, all associations remained statistically significant. In Model 2, women with a history of any IPV had more than twice the prevalence of depressive symptoms compared with women without IPV (aPR = 2.37; 95% CI: 2.18–2.58; *p* < 0.001). Emotional IPV was associated with a 2.32-fold higher prevalence of depressive symptoms (aPR = 2.32; 95% CI: 2.14–2.53; *p* < 0.001), while physical IPV was associated with a 2.21-fold higher prevalence (aPR = 2.21; 95% CI: 2.04–2.39; *p* < 0.001). Sexual IPV was also associated with a higher prevalence of depressive symptoms (aPR = 1.97; 95% CI: 1.80–2.17; *p* < 0.001). Each IPV measure was examined in a separate adjusted model because the different forms of IPV were not mutually exclusive (Table 3). The complete set of Model 2 covariate estimates is presented in Supplementary Table S6.

**Table 3:** Adjusted association between intimate partner violence and depressive symptoms among ever-partnered Peruvian women, ENDES 2015–2024.

IPV measure (models fitted separately)	Crude PR (95% CI)	*p*-value	Model 1 aPR (95% CI)	*p*-value	Model 2 aPR (95% CI)	*p*-value
Any IPV	3.22 (2.99–3.47)	<0.001	2.86 (2.64–3.09)	<0.001	2.37 (2.18–2.58)	<0.001
Emotional IPV	3.19 (2.97–3.42)	<0.001	2.81 (2.60–3.04)	<0.001	2.32 (2.14–2.53)	<0.001
Physical IPV	3.03 (2.82–3.25)	<0.001	2.66 (2.47–2.87)	<0.001	2.21 (2.04–2.39)	<0.001
Sexual IPV	3.17 (2.91–3.45)	<0.001	2.44 (2.22–2.69)	<0.001	1.97 (1.80–2.17)	<0.001

Estimates are weighted using the ENDES health-module sampling weight for persons aged ≥15 years (PESO15_AJUS/1,000,000 in 2015 and PESO15_AMAS/1,000,000 in 2016–2024). All inferential estimates accounted for the complex survey design, including sampling weights, strata, and clustered primary sampling units. PR/aPR were estimated using modified Poisson regression with robust variance. Model 1 adjusted for survey period, age group, educational level, union status, current work status, number of children living in the household, area of residence, natural region, wealth quintile, and mother tongue. Model 2 adjusted for Model 1 covariates plus partner alcohol consumption, controlling behaviors by partner, and father beating mother. Any IPV, emotional IPV, physical IPV, and sexual IPV were entered in separate adjusted models to avoid overadjustment due to overlap between IPV types. IPV was interpreted as history of partner violence by the current or most recent husband/partner rather than incident IPV. Source: ENDES 2015–2024.

The sensitivity analysis excluding partner controlling behaviors while retaining partner alcohol consumption and intergenerational violence yielded estimates intermediate between Models 1 and 2. Any IPV remained associated with a 2.73-fold higher prevalence of depressive symptoms (aPR = 2.73; 95% CI: 2.52–2.96; *p* < 0.001). Corresponding estimates were 2.70 for emotional IPV (95% CI: 2.49–2.92), 2.54 for physical IPV (95% CI: 2.35–2.75), and 2.32 for sexual IPV (95% CI: 2.11–2.55), all *p* < 0.001. Thus, excluding controlling behaviors increased the estimates relative to Model 2 but did not materially alter the direction or statistical interpretation of the associations (Supplementary Table S7).

A complete-case sensitivity analysis was additionally conducted among 93,035 women with complete information for all Model 2 covariates. The estimates were similar to those from the primary analysis that retained missing/not-reported categories: any IPV, aPR = 2.40 (95% CI: 2.20–2.62); emotional IPV, aPR = 2.35 (95% CI: 2.16–2.56); physical IPV, aPR = 2.25 (95% CI: 2.08–2.44); and sexual IPV, aPR = 1.98 (95% CI: 1.80–2.18); all *p* < 0.001. These findings indicate that the handling of missing covariate information did not materially alter the main associations (Supplementary Table S8).

### 3.5 Association between intimate partner violence and depressive symptoms by survey period

The association between any IPV and depressive symptoms remained statistically significant across all three survey periods. During 2015–2019, the weighted prevalence of depressive symptoms was 12.5% among women with a history of IPV and 3.4% among those without IPV. After adjustment, women reporting IPV had 2.72 times the prevalence of depressive symptoms observed among women without IPV (aPR = 2.72; 95% CI: 2.43–3.04; *p* < 0.001).

During 2020–2021, depressive symptoms were reported by 10.7% of women with IPV and 4.3% of those without IPV. The adjusted prevalence ratio during this period was 1.77 (95% CI: 1.47–2.12; *p* < 0.001). During 2022–2024, the weighted prevalence of depressive symptoms was 14.9% among women with IPV and 4.6% among those without IPV, corresponding to an adjusted prevalence ratio of 2.29 (95% CI: 2.00–2.61; *p* < 0.001).

The interaction between any IPV and survey period was statistically significant (global p for interaction <0.001), indicating temporal heterogeneity in the magnitude of the IPV–depressive symptoms association. Pairwise interaction contrasts showed that the adjusted association was weaker in 2020–2021 than in 2015–2019 (ratio of aPRs = 0.65; 95% CI: 0.53–0.80; *p* < 0.001). The association in 2022–2024 was also modestly weaker than in 2015–2019 (ratio of aPRs = 0.84; 95% CI: 0.71–0.99; *p* = 0.038), but was stronger than during 2020–2021 (ratio of aPRs = 1.29; 95% CI: 1.04–1.61; *p* = 0.019; Table 4). The temporal interaction remained evident in both sensitivity analyses. When the interaction model was adjusted using Model 1 covariates only, the period-specific aPRs for any IPV were 3.21 (95% CI: 2.88–3.59) in 2015–2019, 2.17 (95% CI: 1.81–2.59) in 2020–2021, and 2.82 (95% CI: 2.48–3.21) in 2022–2024, with a significant global interaction (*p* < 0.001). The ratio of aPRs for 2020–2021 versus 2015–2019 was 0.68 (95% CI: 0.55–0.83; *p* < 0.001). In the complete-case Model 2 analysis (N = 93,035), the corresponding aPRs were 2.72 (95% CI: 2.43–3.05), 1.81 (95% CI: 1.47–2.23), and 2.27 (95% CI: 1.98–2.60), respectively, and the global interaction remained significant (*p* = 0.001). The 2020–2021 versus 2015–2019 ratio of aPRs was 0.66 (95% CI: 0.53–0.84; *p* < 0.001). Thus, the lower relative association observed during 2020–2021 was not eliminated by restricting adjustment to Model 1 covariates or by complete-case analysis (Supplementary Table S9).

**Table 4:** Association between intimate partner violence and depressive symptoms by survey period, ENDES 2015–2024.

Panel A. Period-specific association between any IPV and depressive symptoms							
Survey period	No IPV, n	Any IPV, n	Depressive symptoms among no IPV (%)	Depressive symptoms among any IPV (%)	Crude PR (95% CI)	Adjusted PR (95% CI)	*p*-value
2015–2019	32,048	19,318	3.4	12.5	3.63 (3.26–4.04)	2.72 (2.43–3.04)	<0.001
2020–2021	12,341	6,849	4.3	10.7	2.48 (2.07–2.96)	1.77 (1.47–2.12)	<0.001
2022–2024	20,144	10,445	4.6	14.9	3.21 (2.83–3.65)	2.29 (2.00–2.61)	<0.001

Panel B. Pairwise contrasts of the adjusted IPV–depressive symptoms association between survey periods		
Pairwise contrast	Ratio of adjusted PRs (95% CI)	*p*-value
2020–2021 vs. 2015–2019	0.65 (0.53–0.80)	<0.001
2022–2024 vs. 2015–2019	0.84 (0.71–0.99)	0.038
2022–2024 vs. 2020–2021	1.29 (1.04–1.61)	0.019

Global p for IPV × survey period interaction: <0.001. N values are unweighted; percentages and regression estimates are survey weighted. IPV, intimate partner violence; PR, prevalence ratio; CI, confidence interval. Adjusted PRs were estimated using survey-weighted modified Poisson regression with robust variance and an IPV × survey period interaction term, adjusting for age group, educational level, union status, current work status, number of children living in the household, area of residence, natural region, wealth quintile, mother tongue, partner alcohol consumption, partner controlling behaviors, and father beating mother. The global interaction p-value was obtained using a design-adjusted Wald test. Pairwise contrasts in Panel B are ratios of the adjusted prevalence ratios for IPV between survey periods; values <1 indicate a weaker IPV–depressive symptoms association in the first listed period relative to the comparison period, whereas values >1 indicate a stronger association. All analyses accounted for the ENDES complex survey design and the health-module sampling weight. Source: ENDES 2015–2024.

## 4 Discussion

Using a linked analytical sample of more than 100,000 ever-partnered Peruvian women derived from 10 consecutive national ENDES surveys, this study found a consistent association between a history of intimate partner violence and depressive symptoms. Women who reported any IPV had more than twice the adjusted prevalence of depressive symptoms compared with those who did not report IPV. Emotional, physical, and sexual IPV also remained associated with depressive symptoms when examined separately, even after accounting for sociodemographic, socioeconomic, geographical, family, and partner-related characteristics. In addition, depressive symptoms showed an overall positive temporal tendency despite substantial year-to-year fluctuations and remained consistently more frequent among women with a history of IPV. Although the strength of the association varied significantly over time and was attenuated during 2020–2021, IPV remained associated with depressive symptoms in every period analyzed. Taken together, these findings indicate that the association between IPV and depressive symptoms was observed across each of the survey periods examined, despite changes in the broader social and temporal context.

The principal adjusted finding was that women with a history of any IPV had more than twice the prevalence of depressive symptoms compared with women who did not report IPV (aPR = 2.37; 95% CI: 2.18–2.58). This estimate was slightly lower than the aPR of 2.47 reported by Silva-Burga et al. (2022) using ENDES 2014–2016 and slightly higher than the aPR of 2.26 observed among pregnant Peruvian women in ENDES 2013–2019 by Benites-Meza et al. (2023). It also falls within the heterogeneous estimates reported by Chaquila et al. (2025), whose wealth-stratified aPRs ranged from 1.37 to 2.71 in ENDES 2023. Beyond Peru, de Baumont et al. (2024) reported aPRs of 1.50 for exposure to one IPV type and 2.70 for exposure to two or three types in Brazil. International meta-analyses found associations with depression ranging from OR 2.04 to 3.14 and a pooled longitudinal OR of 1.92 (White et al., 2024; Watson and Bitsika, 2025).

Although prevalence ratios and odds ratios are not directly interchangeable, the overall pattern is consistent with a substantial cross-sectional association across settings. In the present study, however, IPV represented ever-reported violence by the current or most recent husband/partner, whereas the PHQ-9 assessed depressive symptoms during the preceding 2 weeks; therefore, the observed association should be interpreted as cross-sectional co-occurrence rather than evidence that IPV preceded or caused current depressive symptoms. Differences in magnitude across studies may reflect population composition, IPV definitions and exposure timeframes, depression instruments and thresholds, covariate selection, survey period, and weighting procedures.

Emotional, physical, and sexual IPV were each associated with depressive symptoms when examined in separate adjusted models, with broadly comparable magnitudes across subtypes. These estimates are broadly consistent with the Peruvian analysis of pregnant women by Benites-Meza et al. (2023), who reported aPRs of 2.01 for psychological violence, 2.34 for physical violence, and 2.31 for sexual violence. A particularly relevant finding was that the magnitude of the association for emotional IPV was comparable to that for physical IPV. This pattern accords with the systematic review by Dokkedahl et al. (2022), which found strong associations between psychological IPV and depression and identified emotional/verbal abuse and dominance/isolation as relevant dimensions, although the certainty of the pooled evidence was rated low. Hernández and Ortega (2025) further showed that recent depression in Peruvian women varied according to combined IPV typologies and severity, underscoring that forms of violence frequently coexist. Because IPV types were evaluated in separate models and were not mutually exclusive, these estimates should not be interpreted as mutually independent effects or as evidence that one IPV type is more harmful than another.

The magnitude of the association between any IPV and depressive symptoms varied across survey periods, with a weaker association during 2020–2021 and a higher magnitude again during 2022–2024. Importantly, this attenuation remained evident in the Model 1 interaction sensitivity analysis and in the complete-case Model 2 interaction analysis, indicating that the temporal pattern was not eliminated by excluding the additional contextual covariates or by restricting the analysis to complete cases. The attenuation during 2020–2021 contrasts superficially with evidence that violence reports increased under COVID-19 restrictions. In Peru, Agüero (2021) documented a 48% increase in calls to the national Línea 100 helpline between March and July 2020, while Hernández-Vásquez et al. (2023) identified an increase during strict lockdown followed by a decline after confinement measures were relaxed. A systematic review and meta-analysis similarly found an overall rise in reported domestic violence after lockdowns (Piquero et al., 2021). However, those studies assessed changes in violence incidents or help-seeking, whereas the present analysis estimated the relative prevalence of depressive symptoms between women with and without reported IPV. The lower relative association observed in 2020–2021 could reflect several non-mutually exclusive mechanisms, including genuine temporal heterogeneity in the association, increased depressive symptoms among women without reported IPV, reduced privacy during interviews, differential underreporting or misclassification of IPV, changes in survey fieldwork, or selection into the analytic sample. None of these mechanisms was directly measured in the present study. Therefore, the attenuation observed during 2020–2021 should be interpreted as descriptive temporal heterogeneity rather than as evidence that IPV had a weaker causal effect during the pandemic. Likewise, the higher estimate observed in 2022–2024 should not be attributed to normalization of reporting conditions, service access, or other specific mechanisms without direct evidence.

The estimated annual relative change in depressive symptoms was +2.31% over 2015–2024, summarizing a non-monotonic annual pattern rather than a steady year-to-year increase. This finding extends the pre-pandemic analysis of Villarreal-Zegarra et al. (2020), which found no clear change in depressive symptoms in Peru from 2014 to 2018 when a more stringent PHQ-9 threshold of ≥15 was used. In contrast, an interrupted time-series analysis covering 2014–2021 detected a sustained quarterly increase of 0.17 percentage points in moderate depressive symptoms after the onset of COVID-19 (Villarreal-Zegarra et al., 2023). During the first pandemic stage, Herrera-Añazco et al. (2022) reported depressive symptoms in 46.6% of more than 1.3 million Latin American and Caribbean respondents and found a greater symptom burden among women and non-binary participants; however, that Facebook-based survey used adapted K10 items and is not directly comparable with ENDES. Similarly, approximately one-third of Peruvian adults screened positive using PHQ-9 ≥ 10 during the first national lockdown in an opt-in online survey (Antiporta et al., 2021). Meanwhile, ENDES 2019 data yielded a prevalence of 6.8% among Peruvian adults (Vasquez-Chavesta et al., 2024). Together, these studies support a modest underlying increase interrupted by pronounced fluctuations, while differences in sampling frames, population composition, instruments, thresholds, and pandemic-specific data collection explain why acute-period estimates were substantially higher.

The weighted prevalence of any IPV was 35.3%, with physical IPV reported by 28.0% of women, emotional IPV by 24.5%, and sexual IPV by 6.1%. These estimates were somewhat lower than those reported from ENDES 2015–2017 by Burgos-Muñoz et al. (2021), who found prevalences of 38.7% for any IPV, 31.2% for physical IPV, and 26.8% for psychological IPV, whereas the sexual IPV estimate was similar (6.9%). Other Peruvian studies have produced more variable estimates. Barón-Lozada et al. (2022), using ENDES 2019 and a previous-12-month definition among currently partnered women, reported 40.1% for any IPV, whereas Bendezu-Quispe et al. (2024) reported a past-year prevalence of 16.0% in a different ENDES 2019 analytic sample. Caira-Chuquineyra et al. (2024) found any IPV in 49.6% of women in ENDES 2020, including psychological IPV in 45.8%, physical IPV in 22.2%, and sexual IPV in 4.3%. At the global level, Sardinha et al. (2022) estimated that 27% of ever-partnered women aged 15–49 had experienced lifetime physical or sexual IPV. That estimate is not directly comparable because it excludes emotional IPV and applies a standardized lifetime definition. The wide variation across studies highlights the importance of distinguishing lifetime exposure, previous-12-month exposure, violence by any partner, and violence attributed to the current or most recent partner when interpreting prevalence.

The weighted prevalence of depressive symptoms was 7.1% using the PHQ-9 cutoff of ≥10. This estimate closely matches the 6.98% reported by Silva-Burga et al. (2022) among Peruvian women aged 15–45 years in ENDES 2014–2016 and the 6.8% reported among Peruvian adults in ENDES 2019 by Vasquez-Chavesta et al. (2024), both of which used the PHQ-9. By contrast, Villarreal-Zegarra et al. (2020) reported a two-week prevalence of 2.3% in 2018 using the more restrictive PHQ-9 cutoff of ≥15; therefore, that estimate should not be interpreted as directly equivalent. At the other extreme, Antiporta et al. (2021) found that approximately one-third of adults screened positive at PHQ-9 ≥ 10 during the first national COVID-19 lockdown. That markedly higher estimate arose from an opt-in online survey conducted during an acute crisis and involved adults of both sexes, making direct comparison with the present linked population-based ENDES sample inappropriate. The present prevalence therefore appears plausible for population-based surveillance outside the most acute lockdown period. Comparisons with studies using the CES-D, BDI, SRQ-20, or diagnostic interviews would require even greater caution because those instruments assess different constructs, timeframes, and thresholds. Consistent use of PHQ-9 ≥ 10 strengthens comparability and supports continued surveillance of clinically relevant depressive symptoms among Peruvian women.

This study contributes to the literature by providing a decade-long, population-based assessment of the relationship between IPV and depressive symptoms among ever-partnered Peruvian women using linked data from 10 consecutive national ENDES surveys, while integrating three dimensions that have often been examined separately: overall and type-specific associations, variation across the pre-pandemic, pandemic, and post-acute pandemic periods, and the temporal trend in depressive symptoms across the national ENDES surveys. The association remained evident after adjustment for sociodemographic, socioeconomic, family, and partner-related characteristics, indicating that IPV is an important marker of mental-health vulnerability across heterogeneous population groups and social contexts. Moreover, the analysis shows that emotional IPV warrants explicit recognition alongside physical and sexual IPV and that the strength of the overall association was not temporally constant. Although ENDES uses a probabilistic, stratified, and clustered national sampling design, the present analyses were based on the linked intersection of the health and domestic-violence modules and additionally required valid IPV information and a positive health-module sampling weight. The findings should therefore be interpreted as population-based evidence derived from the national ENDES survey framework rather than as estimates demonstrated to be fully representative of all ever-partnered Peruvian women aged 15–49 years. Generalization to never-partnered women, women outside this age range, men, or clinical populations is not supported by the present data. These results nevertheless provide a contemporary population-based benchmark for future causal, longitudinal, and intervention studies.

From a public health perspective, these findings underscore the importance of recognizing the frequent co-occurrence of IPV and depressive symptoms and of ensuring that health and social-service systems are prepared to respond safely when either problem is identified. Particular attention to emotional IPV may be warranted because its association with depressive symptoms was comparable in magnitude to that observed for physical IPV and it may be less readily recognized in routine care. However, the present observational study did not evaluate the effectiveness of universal IPV screening, routine PHQ-9 implementation, or any specific referral pathway. Accordingly, the findings should not be interpreted as evidence that a particular screening or service-delivery strategy improves clinical outcomes. Clinical and public-health responses should instead follow established evidence-based guidelines, local protocols, and privacy- and safety-sensitive procedures. Continued population surveillance of IPV and depressive symptoms may also be useful given the observed temporal heterogeneity in their association.

The main strengths of this study include the large analytic sample, the use of 10 consecutive nationally representative ENDES surveys, and appropriate incorporation of sampling weights, strata, and primary sampling units within the health-module sampling framework, which improved the precision and population-based relevance of the estimates. The use of a standardized PHQ-9 cutoff permitted consistent measurement of depressive symptoms across survey years, while modified Poisson regression provided directly interpretable prevalence ratios. Separate models for any, emotional, physical, and sexual IPV avoided simultaneous adjustment for overlapping violence types, and the period-specific models and interaction analysis allowed temporal heterogeneity in the IPV–depressive symptoms association to be formally evaluated.

Several limitations should also be acknowledged. The repeated cross-sectional design precludes establishing temporality or causality, and reverse or bidirectional relationships cannot be excluded. This limitation is particularly important because IPV represented ever-reported violence by the current or most recent husband/partner, whereas the PHQ-9 assessed depressive symptoms during the preceding 2 weeks; therefore, the study demonstrates cross-sectional co-occurrence rather than establishing that IPV preceded or caused current depressive symptoms. IPV and depressive symptoms were self-reported and may be affected by recall, social-desirability, and underreporting biases. The IPV measures did not fully capture the timing, frequency, duration, severity, or violence perpetrated by all previous partners. The PHQ-9 identifies clinically relevant depressive symptoms but does not establish a psychiatric diagnosis. Because IPV types may coexist, the separate subtype models cannot determine their mutually independent effects.

Additional limitations relate to participant selection, module eligibility, privacy requirements, and survey weighting. Inclusion in the primary analysis required records to be available in both the health and domestic-violence modules, complete PHQ-9 information, sufficient valid IPV information, and a positive health-module sampling weight. Women excluded because of incomplete PHQ-9 or IPV information differed descriptively from those included, particularly with respect to age, union status, and urban residence, indicating that some selection bias cannot be excluded. In addition, ENDES administers the domestic-violence section only when adequate privacy can be ensured; consequently, women for whom privacy-related administration conditions were not met may be underrepresented in the IPV analytic sample. If inability to obtain privacy is associated with partner control or IPV risk, both selection bias and underreporting are possible, and the harmonized data did not allow privacy-related non-administration to be separately quantified across all survey years. Finally, the primary sampling weight was the health-module weight rather than a joint health–domestic-violence weight because ENDES does not provide a consistent official weight for the intersection of these two subsamples across all survey rounds. Therefore, residual selection related to participation in the domestic-violence module may not have been fully corrected by weighting.

Further limitations concern covariate adjustment, missing data, changes in survey administration, temporal modeling, and generalizability. Residual confounding and overadjustment remain possible because the temporal ordering of IPV relative to union status, employment, number of children, and partner controlling behaviors cannot be established. Missing/not-reported categories were retained for selected covariates to preserve observations in the primary analysis; however, this approach does not eliminate bias when missingness is informative and the coefficients for these categories have no substantive epidemiological interpretation. Missing covariate information was strongly concentrated in 2020 and, to a lesser extent, 2021.

Although the overall IPV estimates and the IPV × survey period interaction remained materially similar in sensitivity analyses using the Model 1 adjustment set and complete cases, bias related to non-random missingness cannot be excluded. ENDES fieldwork also changed during 2020–2021, including the use of telephone interviewing and modified field procedures, and the harmonized data did not provide a consistently comparable individual-level indicator of interview mode or privacy across all years. These changes may have affected reporting, exposure classification, or sample composition and therefore complicate interpretation of temporal differences. The continuous-year log-linear model summarizes an average temporal tendency and cannot capture all nonlinear year-to-year variation; this limitation was addressed by presenting year-specific prevalence estimates and additionally evaluating survey year as a categorical variable. Finally, the findings apply to women aged 15–49 years who had ever been married or in a cohabiting union and should not be generalized to never-partnered women, women outside this age range, or women whose only intimate relationships were non-cohabiting dating relationships.

Among ever-partnered Peruvian women surveyed between 2015 and 2024, a history of IPV was consistently associated with a substantially higher prevalence of depressive symptoms. Emotional, physical, and sexual IPV were each associated with the outcome after adjustment, reinforcing that less visible forms of violence should not be treated as clinically secondary. The association was observed in each survey period, although its magnitude differed, with a lower estimate during 2020–2021 and a higher estimate again during 2022–2024. Depressive symptoms also showed a modest positive annual relative change over the study period, superimposed on marked year-to-year fluctuations. These findings do not establish that IPV caused depressive symptoms, but they demonstrate a population-based co-occurrence within the linked ENDES analytical sample that remained evident after adjustment. They support greater clinical and public-health attention to co-occurring IPV and depressive symptoms but do not establish the effectiveness of universal IPV screening, routine PHQ-9 implementation, or any specific referral pathway. Longitudinal and intervention research is needed to clarify temporal pathways, cumulative exposure, recurrence, and the effectiveness of strategies for identification, mental-health assessment, and referral before, during, and after major social disruptions such as the COVID-19 pandemic.

## Data Availability

Publicly available datasets were analyzed in this study. This data can be found at: the original ENDES microdata are publicly available from the National Institute of Statistics and Informatics of Peru (INEI) through its Microdata Repository: https://iinei.inei.gob.pe/microdatos/. No accession number is assigned to these annual survey datasets.
