# Supplementary material for: Intimate partner violence and depressive symptoms among ever-partnered Peruvian women: a repeated cross-sectional analysis of national surveys, 2015–2024
Source: Front Psychol. 2026 Oct 14;17:1939992. doi: 10.3389/fpsyg.2026.1939992 (PMC13713516; doi:10.3389/fpsyg.2026.1939992)
Supplement: Supplementary file 1 [file Supplementary_file_1.doc]

**Table S1.** Sample selection and missing-data patterns, ENDES 2015–2024

**Panel A.** Year-specific analytic sample selection

| Year | Women in REC0111 | Records available in linked health + domestic-violence modules | Ever-partnered women | Complete PHQ-9 | Complete IPV information | Final sample with positive health-module weight |
| --- | --- | --- | --- | --- | --- | --- |
| 2015 | 36,655 | 13,896 | 11,350 | 11,347 | 10,109 | 10,109 |
| 2016 | 34,131 | 13,533 | 11,063 | 11,063 | 9,922 | 9,888 |
| 2017 | 34,002 | 13,877 | 11,396 | 11,396 | 10,257 | 10,237 |
| 2018 | 39,745 | 14,631 | 12,092 | 12,092 | 10,852 | 10,827 |
| 2019 | 38,335 | 14,024 | 11,471 | 11,471 | 10,352 | 10,305 |
| 2020 | 37,895 | 11,357 | 9,193 | 9,193 | 8,665 | 8,579 |
| 2021 | 38,635 | 14,021 | 11,609 | 11,609 | 10,613 | 10,611 |
| 2022 | 38,105 | 13,811 | 11,344 | 11,344 | 10,273 | 10,268 |
| 2023 | 38,352 | 13,869 | 11,401 | 11,401 | 10,392 | 10,384 |
| 2024 | 37,117 | 13,408 | 10,993 | 10,993 | 9,941 | 9,937 |
| Total | **372,972** | **136,427** | **111,912** | **111,909** | **101,376** | **101,145** |

**Note:** Counts are unweighted and represent sequential stages of sample construction. The difference between the complete women’s file (REC0111) and the linked health + domestic-violence analytic file primarily reflects module-specific sampling, eligibility, and availability of records in both required modules and should not be interpreted solely as item nonresponse or linkage failure. Among the 136,427 women with records available in both modules, 24,515 were not ever-partnered and were therefore ineligible for the present analysis. Among ever-partnered women, 3 had incomplete PHQ-9 information and an additional 10,533 did not have sufficient valid IPV information, yielding 101,376 women who met the substantive analytic criteria. Of these, 231 were excluded from weighted estimation because the health-module sampling weight was missing (n=197) or zero (n=34), resulting in a primary weighted analytic sample of 101,145 women.

**Panel B.** Year-specific missingness in covariates retained with explicit missing/not-reported categories

| Year | Analytic N | Current work status missing, n (%) | Partner alcohol missing, n (%) | Controlling behaviors missing, n (%) | Father beat mother missing, n (%) | ≥1 of four covariates missing, n (%) |
| --- | --- | --- | --- | --- | --- | --- |
| 2015 | 10,109 | 3 (<0.1) | 2 (<0.1) | 2 (<0.1) | 372 (3.7) | 377 (3.7) |
| 2016 | 9,888 | 0 (0.0) | 0 (0.0) | 1 (<0.1) | 353 (3.6) | 354 (3.6) |
| 2017 | 10,237 | 0 (0.0) | 0 (0.0) | 1 (<0.1) | 374 (3.7) | 374 (3.7) |
| 2018 | 10,827 | 0 (0.0) | 0 (0.0) | 7 (0.1) | 401 (3.7) | 408 (3.8) |
| 2019 | 10,305 | 0 (0.0) | 0 (0.0) | 3 (<0.1) | 390 (3.8) | 392 (3.8) |
| 2020 | 8,579 | 3,890 (45.3) | 2,473 (28.8) | 2 (<0.1) | 327 (3.8) | 4,043 (47.1) |
| 2021 | 10,611 | 787 (7.4) | 47 (0.4) | 7 (0.1) | 383 (3.6) | 1,150 (10.8) |
| 2022 | 10,268 | 0 (0.0) | 0 (0.0) | 2 (<0.1) | 382 (3.7) | 384 (3.7) |
| 2023 | 10,384 | 0 (0.0) | 0 (0.0) | 2 (<0.1) | 336 (3.2) | 338 (3.3) |
| 2024 | 9,937 | 0 (0.0) | 0 (0.0) | 3 (<0.1) | 288 (2.9) | 290 (2.9) |
| Total | **101,145** | **4,680 (4.6)** | **2,522 (2.5)** | **30 (<0.1)** | **3,606 (3.6)** | **8,110 (8.0)** |

**Note:** Counts and percentages in Panel B are unweighted and were calculated within the primary weighted analytic sample (N=101,145). Missingness was defined using the original observed status of each covariate before creation of explicit missing/not-reported categories for regression modeling. Because missingness could occur in more than one covariate for the same woman, the four covariate-specific columns are not mutually exclusive and should not be summed. The final column identifies women with at least one missing value across current work status, partner alcohol consumption, partner controlling behaviors, or father beating mother.

**Panel C.** Descriptive comparison of women included in and excluded from the primary analytic sample

| Characteristic | Included in primary analysis, N=101,145, weighted % | Excluded because of incomplete PHQ-9/IPV information, N=10,493, weighted % |
| --- | --- | --- |
| Age group |  |  |
| 15–19 years | 3.3 | 5.7 |
| 20–29 years | 31.6 | 23.2 |
| 30–39 years | 38.0 | 24.0 |
| 40–49 years | 27.0 | 47.1 |
| Educational level |  |  |
| No formal/initial | 1.7 | 1.9 |
| Primary | 18.6 | 20.8 |
| Secondary | 45.6 | 46.9 |
| Higher | 34.2 | 30.3 |
| Union status |  |  |
| Currently married/cohabiting | 81.8 | 71.3 |
| Formerly in union | 18.2 | 28.7 |
| Area of residence |  |  |
| Urban | 77.4 | 83.6 |
| Rural | 22.6 | 16.4 |
| Natural region |  |  |
| Coast | 25.7 | 27.8 |
| Highlands | 26.8 | 21.5 |
| Rainforest | 14.8 | 13.3 |
| Metropolitan Lima | 32.8 | 37.3 |
| Wealth quintile |  |  |
| Poorest | 20.9 | 14.4 |
| Poorer | 24.0 | 20.4 |
| Middle | 21.5 | 23.3 |
| Richer | 18.6 | 23.0 |
| Richest | 15.0 | 18.9 |

**Note:** Panel C was restricted to ever-partnered women with a positive health-module sampling weight and records available in the linked health and domestic-violence module intersection. Included women had complete PHQ-9 information and sufficient valid IPV information and therefore entered the primary analysis (N=101,145). Excluded women had incomplete PHQ-9 information (n=3) or insufficient valid IPV information (n=10,490), yielding N=10,493. Percentages are weighted using the ENDES health-module sampling weight and are presented descriptively to characterize potential selection; no hypothesis tests were performed because the purpose was not to test equivalence between included and excluded women. Weighted percentages may not total exactly 100% because of rounding.

**Abbreviations:** ENDES, Demographic and Family Health Survey; PHQ-9, Patient Health Questionnaire-9; IPV, intimate partner violence.

**Table S2.** Variables used, operational definitions, source variables, and recoding rules

| Manuscript variable | Operational definition | Recoding rule | ENDES source / harmonized variable(s) | Final categories | Reference category |
| --- | --- | --- | --- | --- | --- |
| Depressive symptoms | Clinically relevant depressive symptoms during the preceding 2 weeks, assessed with the nine-item Patient Health Questionnaire (PHQ-9). | Original QS700A–QS700I responses were scored 0–3 and summed to a total score of 0–27. All nine items were required to be valid. PHQ-9 ≥10 was coded as depressive symptoms and PHQ-9 <10 as no depressive symptoms. | Original ENDES health-module variables QS700A–QS700I; harmonized as phq_item1–phq_item9; derived phq9_score and depressive_symptoms. | No depressive symptoms; Depressive symptoms | No depressive symptoms |
| Any intimate partner violence | Ever-reported emotional, physical, or sexual IPV perpetrated by the current or most recent husband/partner. | Author-derived composite coded 1 when ≥1 of ipv_emotional, ipv_physical, or ipv_sexual was positive; coded 0 when all three were negative. Missing/non-applicable IPV information was not recoded as absence of violence. | Derived from ipv_emotional, ipv_physical, and ipv_sexual; final variable ipv_any | No IPV; Any IPV | No IPV |
| Emotional IPV | Ever-reported emotional violence perpetrated by the current or most recent husband/partner, as summarized by the original ENDES variable D104. | D104=1 → Yes; D104=0 → No. Original missing/non-applicable values remained missing. | Original ENDES D104; harmonized copy raw_d104; derived analytic variable ipv_emotional | No; Yes | No |
| Physical IPV | Ever-reported less-severe or severe physical violence perpetrated by the current or most recent husband/partner. | Coded 1 when D106=1 or D107=1; coded 0 when D106=0 and D107=0. Original missing/non-applicable information was not automatically recoded as No. | Original ENDES D106 (less-severe physical violence) and D107 (severe physical violence); harmonized copies raw_d106, raw_d107; derived analytic variable ipv_physical | No; Yes | No |
| Sexual IPV | Ever-reported sexual violence perpetrated by the current or most recent husband/partner, as summarized by the original ENDES variable D108. | D108=1 → Yes; D108=0 → No. Original missing/non-applicable values remained missing. | Original ENDES D108; harmonized copy raw_d108; derived analytic variable ipv_sexual | No; Yes | No |
| Survey period | Analytical calendar period based on survey year. | Survey years were grouped as 2015–2019, 2020–2021, and 2022–2024. | anio; derived period | 2015–2019; 2020–2021; 2022–2024 | 2015–2019 |
| Age group | Woman’s age at interview. | Categorized as 15–19, 20–29, 30–39, and 40–49 years. | age; age_group | 15–19; 20–29; 30–39; 40–49 years | 20–29 years |
| Educational level | Highest educational level attained by the woman. | Harmonized into no formal/initial, primary, secondary, and higher education. | educ_attain; educ_level | No formal/initial; Primary; Secondary; Higher | Higher |
| Union status | Current union status among women who had ever been married or in a cohabiting union. | Categorized as currently married/cohabiting or formerly in union. | marital_status; union_status | Currently married/cohabiting; Formerly in union | Currently married/cohabiting |
| Currently working | Current employment status at the time of the survey. | Coded as No, Yes, or Missing/not reported. | currently_working; worked_last12mo | No; Yes; Missing/not reported | No |
| Number of children living in household | Number of children living in the woman’s household. | Categorized as 0, 1–2, and ≥3 children. | n_children_living_hh; derived children_cat | 0; 1–2; ≥3 | 0 |
| Area of residence | Urban or rural area of residence. | Original ENDES residence category harmonized as urban or rural. | area | Urban; Rural | Urban |
| Natural region | Natural region of residence. | Harmonized as Coast, Highlands, Rainforest, and Metropolitan Lima. | raw_sregion; region_nat | Coast; Highlands; Rainforest; Metropolitan Lima | Coast |
| Wealth quintile | Household wealth quintile derived by ENDES. | Retained according to the five original ENDES wealth categories. | wealth_quintile | Poorest; Poorer; Middle; Richer; Richest | Richest |
| Mother tongue | Woman’s reported mother tongue/language, harmonized for analysis. | Categorized as Spanish/Castilian, Indigenous language, and Other/not reported. | raw_s119; mother_tongue; derived mother_tongue_cat | Spanish/Castilian; Indigenous language; Other/not reported | Spanish/Castilian |
| Partner drinks alcohol | Reported alcohol consumption by the current or most recent husband/partner. | Coded as No, Yes, or Missing/not reported. | Original ENDES field preserved as raw_d113; derived partner_drinks_alcohol; analytic partner_alcohol_cat | No; Yes; Missing/not reported | No |
| Controlling behaviors by partner | Presence of at least one reported controlling behavior by the current or most recent husband/partner. | Coded 1 when ≥1 controlling behavior was reported and 0 when none was reported; missing information was retained separately. | control_behaviors_count; derived control_behaviors_any; analytic control_cat | No; Yes; Missing/not reported | No |
| Father beat mother | Woman reported that her father had physically assaulted her mother. | Coded as No, Yes, or Missing/not reported. | Original ENDES field preserved as raw_d121; derived father_beat_mother; analytic father_beat_cat | No; Yes; Missing/not reported | No |
| Sampling weight | ENDES health-module sampling weight for the selected household member aged ≥15 years. | Original factor divided by 1,000,000. PESO15_AJUS was used in 2015 and PESO15_AMAS in 2016–2024. Observations with missing or zero health-module weights were excluded from weighted estimation. | PESO15_AJUS (2015); PESO15_AMAS (2016–2024); harmonized weight_health; analytic weight_analysis | Continuous sampling weight | Not applicable |

Note: IPV measures represent ever-reported violence perpetrated by the current or most recent husband/partner and should not be interpreted as incident IPV or as IPV occurring exclusively during the previous 12 months. D104, D106, D107, and D108 are original ENDES domestic-violence summary variables. Specifically, D106 summarizes less-severe physical violence based on D105A–D105D, D107 summarizes severe physical violence based on D105E–D105G, and D108 summarizes sexual violence based on D105H–D105I. The raw_ prefix was assigned by the authors during harmonization to preserve a copy of the corresponding original ENDES field before derivation of the final analytical variable; therefore, raw_d104, raw_d106, raw_d107, and raw_d108 are harmonized copies rather than original ENDES variable names or newly constructed IPV measures. Original missing or non-applicable IPV values were not recoded as absence of violence. Missing/not reported categories were retained for current work status, partner alcohol consumption, controlling behaviors, and father beating mother. Depressive symptoms were defined as PHQ-9 score ≥10. Primary weighted analyses used the ENDES health-module sampling weight for persons aged ≥15 years (PESO15_AJUS in 2015 and PESO15_AMAS in 2016–2024), divided by 1,000,000. Weighted percentages may not total exactly 100% because of rounding.

**Table S3.** Detailed operationalization and harmonization of intimate partner violence measures across ENDES 2015–2024

| Analytic IPV measure | Original ENDES source variable(s) | Questionnaire content / source items | Positive-response definition used in the analysis | Exposure timeframe and partner reference | Missing/non-applicable handling | Harmonization across 2015–2024 |
| --- | --- | --- | --- | --- | --- | --- |
| Emotional IPV | D104 | D104 is the official ENDES binary summary variable for emotional violence. The associated emotional-violence block includes D103A (humiliation in front of others), D103B (threatened harm to the woman or someone close), D103C (insulted or made her feel bad), D103D (threatened to leave, take the children, or withdraw economic support), together with country-specific emotional-abuse fields D103E–D103F where present. | D104=1 → Emotional IPV; D104=0 → No emotional IPV. D104 was used directly; the authors did not reconstruct this summary variable from D103 items. | Ever-reported emotional violence by the current or most recent husband/partner. Subsequent questions on frequency during the previous 12 months were not used to define the exposure. | Original missing or not-applicable values were retained as missing and were not recoded as No. | D104 was present with binary coding 0=No and 1=Yes throughout 2015–2024. The field was copied into raw_d104, from which ipv_emotional was defined. The wording of the emotional-violence block was reviewed by year; no change in the D104 binary coding was identified. |
| Less-severe physical IPV | D106, derived by ENDES from D105A–D105D | D105A: pushed, shook, or threw something at her. D105B: slapped her in the earlier rounds; from 2019 onward, slapped her or twisted her arm. D105C: hit her with a fist or an object that could cause harm. D105D: kicked or dragged her. | D106=1 → At least one less-severe physical IPV act; D106=0 → No less-severe physical IPV. D106 was used directly rather than reconstructed by the authors. | Ever-reported physical violence by the current or most recent husband/partner. Previous-12-month frequency responses were not used in the exposure definition. | Original missing/not-applicable values remained missing. | D106 remained the official binary summary of D105A–D105D across all rounds, with 0=No and 1=Yes. A wording change was identified for D105B: the earlier surveys referred to slapping, whereas from 2019 onward the item referred to slapping or twisting the arm. This was not recoded manually because the official D106 summary variable was retained. D106 was copied into raw_d106. |
| Severe physical IPV | D107, derived by ENDES from D105E–D105G | D105E: attempted to strangle or burn her. D105F: threatened her with a knife, firearm, or another weapon. D105G: attacked her with a knife, firearm, or another weapon. | D107=1 → At least one severe physical IPV act; D107=0 → No severe physical IPV. D107 was used directly. | Ever-reported severe physical violence by the current or most recent husband/partner. Previous-12-month frequency responses were not used. | Original missing/not-applicable values remained missing. | D107 retained the same binary definition based on D105E–D105G across the study period. It was copied into raw_d107. No manual reconstruction of D107 was performed. |
| Physical IPV | Author-derived from D106 and D107 | Combined ENDES less-severe and severe physical IPV summary measures. | ipv_physical=1 when D106=1 **or** D107=1; ipv_physical=0 when both D106=0 and D107=0. A missing source value was not automatically interpreted as absence of violence. | Ever-reported physical IPV by the current or most recent husband/partner. | Women without sufficient valid source information to classify physical IPV were considered to have incomplete IPV information rather than being assigned to the unexposed category. | The same derivation was applied after annual harmonization. Original D106 and D107 were preserved as raw_d106 and raw_d107; the final derived variable was ipv_physical. |
| Sexual IPV | D108, derived by ENDES from D105H–D105I | D105H: current or most recent husband/partner used physical force to make the woman have sexual intercourse when she did not want to. D105I: partner forced her to perform sexual acts that she did not approve of. | D108=1 → Sexual IPV; D108=0 → No sexual IPV. D108 was used directly rather than reconstructed from D105H–D105I. | Ever-reported sexual violence by the current or most recent husband/partner. Previous-12-month frequency responses were not used. | Original missing/not-applicable values remained missing. | D108 remained the official binary summary of D105H–D105I across 2015–2024, with 0=No and 1=Yes. It was copied into raw_d108, from which ipv_sexual was defined. |
| Any IPV | Author-derived from ipv_emotional, ipv_physical, and ipv_sexual | Composite of emotional, physical, and sexual IPV as defined above. | ipv_any=1 if ≥1 IPV subtype was positive; ipv_any=0 when emotional, physical, and sexual IPV were all negative. | Ever-reported IPV by the current or most recent husband/partner. | Missing/non-applicable source responses were not automatically converted to No. Women without sufficient valid IPV information were classified as having incomplete IPV information and were not included in the analytic IPV sample. | The identical composite rule was applied to all ten survey rounds after harmonization of the original ENDES summary variables. |

**Note:** D104, D106, D107, and D108 are original ENDES domestic-violence summary variables and were not created by the authors. ENDES defines D106 as any less-severe physical violence based on D105A–D105D, D107 as any severe physical violence based on D105E–D105G, and D108 as any sexual violence based on D105H–D105I. The raw_ prefix was assigned during harmonization to preserve a standardized copy of each original ENDES variable before construction of the final analytic variables. Thus, raw_d104, raw_d106, raw_d107, and raw_d108 are author-assigned harmonized field names, not original ENDES variable names and not independently constructed violence measures. The annual questionnaires and data dictionaries were reviewed before pooling. D104, D106, D107, and D108 retained their original binary coding throughout 2015–2024. A wording change was identified for D105B: the earlier rounds referred to slapping, whereas from 2019 onward the item referred to slapping or twisting the arm. This difference was documented rather than manually recoded because the official ENDES D106 summary measure was used in every survey year. Original missing and non-applicable IPV responses were never automatically classified as absence of violence. IPV measures represent ever-reported violence by the current or most recent husband/partner; previous-12-month frequency responses were not used to define the exposures.

**Table S4.** Multicollinearity diagnostics for variables included in Model 2

| Covariate | df | GVIF^(1/[2×df]) |
| --- | --- | --- |
| Age group | 3 | 1.05 |
| Area of residence | 1 | 1.42 |
| Number of children in household | 2 | 1.05 |
| Controlling behaviors | 2 | 1.06 |
| Educational level | 3 | 1.10 |
| Father beat mother | 2 | 1.01 |
| Any intimate partner violence | 1 | 1.14 |
| Mother tongue | 2 | 1.06 |
| Partner drinks alcohol | 2 | 1.21 |
| Survey period | 2 | 1.10 |
| Natural region | 3 | 1.07 |
| Union status | 1 | 1.08 |
| Wealth quintile | 4 | 1.14 |
| Currently working | 2 | 1.27 |

Note: GVIF, generalized variance inflation factor; df, degrees of freedom. Because several predictors were categorical and represented by more than one model parameter, the degrees-of-freedom-adjusted quantity GVIF^(1/[2×df]) is reported to facilitate comparison across terms of different dimensionality. Values close to 1 indicate limited numerical collinearity; the maximum adjusted GVIF was 1.42. These diagnostics assess numerical collinearity only and do not rule out conceptual overadjustment, mediation, or uncertainty in the temporal ordering of covariates.

**Table S5.** Year-specific weighted prevalence of depressive symptoms and categorical-year sensitivity analysis, ENDES 2015–2024

| Year | N | Overall depressive symptoms (%) | No IPV (%) | Any IPV (%) | Categorical-year PR (95% CI) | p-value |
| --- | --- | --- | --- | --- | --- | --- |
| 2015 | 10,109 | 7.5 | 3.7 | 13.6 | Reference | — |
| 2016 | 9,888 | 6.9 | 3.8 | 12.2 | 0.92 (0.80–1.07) | 0.272 |
| 2017 | 10,237 | 6.1 | 2.9 | 11.9 | 0.82 (0.70–0.95) | 0.009 |
| 2018 | 10,827 | 6.1 | 3.0 | 11.8 | 0.82 (0.70–0.95) | 0.010 |
| 2019 | 10,305 | 7.2 | 3.9 | 13.0 | 0.97 (0.81–1.14) | 0.680 |
| 2020 | 8,579 | 5.7 | 3.7 | 9.4 | 0.76 (0.64–0.90) | 0.001 |
| 2021 | 10,611 | 7.3 | 4.9 | 11.9 | 0.97 (0.83–1.13) | 0.679 |
| 2022 | 10,268 | 7.6 | 4.2 | 13.8 | 1.01 (0.87–1.17) | 0.917 |
| 2023 | 10,384 | 8.4 | 4.9 | 15.6 | 1.12 (0.97–1.30) | 0.122 |
| 2024 | 9,937 | 8.2 | 4.8 | 15.4 | 1.10 (0.94–1.28) | 0.231 |

Note: N values are unweighted; prevalence estimates are weighted using the ENDES health-module sampling weight for persons aged ≥15 years (PESO15_AJUS/1,000,000 in 2015 and PESO15_AMAS/1,000,000 in 2016–2024) and account for the complex survey design. Depressive symptoms were defined as a PHQ-9 score ≥10. IPV was interpreted as history of partner violence perpetrated by the current or most recent husband/partner rather than incident IPV. For the categorical-year sensitivity analysis, survey year was entered as a categorical predictor in an unadjusted survey-weighted generalized linear model with a Poisson family and log link, using 2015 as the reference year. PRs represent prevalence ratios for depressive symptoms relative to 2015 and are reported with 95% confidence intervals. The overall design-adjusted Wald test indicated statistically significant differences across survey years (p<0.001). This sensitivity analysis was conducted to assess temporal variation without assuming a constant log-linear change across years.

Source: ENDES 2015–2024.

**Table S6.** Full adjusted model for depressive symptoms among ever-partnered Peruvian women, ENDES 2015–2024

| Associated factors | Adjusted PR (95% CI) | p-value |
| --- | --- | --- |
| Any intimate partner violence |  |  |
| No IPV | Reference |  |
| Any IPV | 2.37 (2.18–2.58) | <0.001 |
| Survey period |  |  |
| 2015–2019 | Reference |  |
| 2020–2021 | 1.09 (0.97–1.22) | 0.154 |
| 2022–2024 | 1.30 (1.20–1.41) | <0.001 |
| Age group |  |  |
| 15–19 years | 1.13 (0.89–1.43) | 0.330 |
| 20–29 years | Reference |  |
| 30–39 years | 1.06 (0.97–1.16) | 0.222 |
| 40–49 years | 1.19 (1.08–1.32) | <0.001 |
| Educational level |  |  |
| No formal/initial | 1.36 (1.08–1.72) | 0.010 |
| Primary | 1.36 (1.20–1.54) | <0.001 |
| Secondary | 1.26 (1.15–1.39) | <0.001 |
| Higher | Reference |  |
| Union status |  |  |
| Currently married/cohabiting | Reference |  |
| Formerly in union | 1.24 (1.14–1.36) | <0.001 |
| Currently working |  |  |
| No | Reference |  |
| Yes | 1.01 (0.93–1.09) | 0.829 |
| Missing/not reported | 0.94 (0.71–1.23) | 0.644 |
| Number of children living in household |  |  |
| 0 | Reference |  |
| 1–2 | 0.77 (0.68–0.87) | <0.001 |
| ≥3 | 0.83 (0.73–0.95) | 0.007 |
| Area of residence |  |  |
| Urban | Reference |  |
| Rural | 0.81 (0.73–0.89) | <0.001 |
| Natural region |  |  |
| Coast | Reference |  |
| Highlands | 1.16 (1.06–1.25) | <0.001 |
| Rainforest | 0.86 (0.78–0.95) | 0.002 |
| Metropolitan Lima | 1.07 (0.96–1.18) | 0.202 |
| Wealth quintile |  |  |
| Poorest | 0.98 (0.80–1.19) | 0.800 |
| Poorer | 1.05 (0.89–1.24) | 0.588 |
| Middle | 1.06 (0.91–1.24) | 0.452 |
| Richer | 1.11 (0.94–1.30) | 0.223 |
| Richest | Reference |  |
| Mother tongue |  |  |
| Spanish/Castilian | Reference |  |
| Indigenous language | 1.07 (0.99–1.16) | 0.072 |
| Other/not reported | 1.85 (0.48–7.18) | 0.374 |
| Partner drinks alcohol |  |  |
| No | Reference |  |
| Yes | 0.99 (0.90–1.08) | 0.806 |
| Missing/not reported | 0.81 (0.56–1.17) | 0.265 |
| Controlling behaviors by partner |  |  |
| No | Reference |  |
| Yes | 1.53 (1.40–1.67) | <0.001 |
| Missing/not reported | 0.44 (0.06–3.33) | 0.428 |
| Father beat mother |  |  |
| No | Reference |  |
| Yes | 1.30 (1.21–1.39) | <0.001 |
| Missing/not reported | 1.31 (1.09–1.57) | 0.004 |

Note: Adjusted PRs were estimated using modified Poisson regression with robust variance and the ENDES health-module sampling weight for persons aged ≥15 years (PESO15_AJUS/1,000,000 in 2015 and PESO15_AMAS/1,000,000 in 2016–2024). All estimates accounted for the complex survey design, including sampling weights, strata, and clustered primary sampling units. The weighted analytic sample comprised 101,145 women. Depressive symptoms were defined as PHQ-9 score ≥10. Missing/not-reported categories were included to retain observations in the primary model; their coefficients are nuisance parameters and were not substantively interpreted.

Source: ENDES 2015–2024.

**Table S7.** Sensitivity analysis of the association between intimate partner violence and depressive symptoms excluding partner controlling behaviors

| IPV measure | Model 1 aPR (95% CI) | Sensitivity model excluding controlling behaviors aPR (95% CI) | Model 2 aPR (95% CI) |
| --- | --- | --- | --- |
| Any IPV | 2.86 (2.64–3.09) | **2.73 (2.52–2.96)** | 2.37 (2.18–2.58) |
| Emotional IPV | 2.81 (2.60–3.04) | **2.70 (2.49–2.92)** | 2.32 (2.14–2.53) |
| Physical IPV | 2.66 (2.47–2.87) | **2.54 (2.35–2.75)** | 2.21 (2.04–2.39) |
| Sexual IPV | 2.44 (2.22–2.69) | **2.32 (2.11–2.55)** | 1.97 (1.80–2.17) |

**Note:** All IPV measures were modeled separately. Model 1 adjusted for survey period, age group, educational level, union status, current work status, number of children living in the household, area of residence, natural region, wealth quintile, and mother tongue. The sensitivity model additionally adjusted for partner alcohol consumption and father beating mother but excluded partner controlling behaviors because controlling behaviors may conceptually overlap with emotional IPV or lie on the exposure pathway. Model 2 additionally included partner alcohol consumption, partner controlling behaviors, and father beating mother. All estimates were obtained using modified Poisson regression with robust variance and accounted for the ENDES complex survey design and health-module sampling weight. All reported IPV associations had p<0.001. aPR: adjusted prevalence ratio; CI: confidence interval; IPV: intimate partner violence.

**Table S8.** Complete-case sensitivity analysis of the association between intimate partner violence and depressive symptoms

| IPV measure | Primary Model 2, N=101,145 aPR (95% CI) | Complete-case Model 2, N=93,035 aPR (95% CI) |
| --- | --- | --- |
| Any IPV | 2.37 (2.18–2.58) | **2.40 (2.20–2.62)** |
| Emotional IPV | 2.32 (2.14–2.53) | **2.35 (2.16–2.56)** |
| Physical IPV | 2.21 (2.04–2.39) | **2.25 (2.08–2.44)** |
| Sexual IPV | 1.97 (1.80–2.17) | **1.98 (1.80–2.18)** |

**Note:** IPV measures were modeled separately. The primary Model 2 retained explicit missing/not-reported categories for current work status, partner alcohol consumption, partner controlling behaviors, and father beating mother. The complete-case sensitivity analysis excluded women with missing information for any covariate included in Model 2. Both models adjusted for survey period, age group, educational level, union status, current work status, number of children living in the household, area of residence, natural region, wealth quintile, mother tongue, partner alcohol consumption, partner controlling behaviors, and father beating mother. Estimates were obtained using modified Poisson regression with robust variance and accounted for the ENDES complex survey design and health-module sampling weight. All IPV associations had p<0.001. aPR: adjusted prevalence ratio; CI: confidence interval; IPV: intimate partner violence. Coefficients for explicit missing/not-reported categories in the primary model were nuisance parameters used to retain observations and were not interpreted as epidemiological associations. Multiple imputation was not performed because the strongly year-dependent missingness pattern did not support a confidently defensible missing-at-random assumption across the pooled survey rounds.

**Table S9. Sensitivity analyses of the temporal interaction between any intimate partner violence and depressive symptoms, ENDES 2015–2024**

| Survey period / contrast | Primary Model 2, N=101,145 aPR (95% CI) | Model 1 sensitivity, N=101,145 aPR (95% CI) | Complete-case Model 2, N=93,035 aPR (95% CI) |
| --- | --- | --- | --- |
| 2015–2019 | 2.72 (2.43–3.04) | 3.21 (2.88–3.59) | 2.72 (2.43–3.05) |
| 2020–2021 | 1.77 (1.47–2.12) | 2.17 (1.81–2.59) | 1.81 (1.47–2.23) |
| 2022–2024 | 2.29 (2.00–2.61) | 2.82 (2.48–3.21) | 2.27 (1.98–2.60) |
| 2020–2021 vs 2015–2019, ratio of aPRs | **0.65 (0.53–0.80)** | **0.68 (0.55–0.83)** | **0.66 (0.53–0.84)** |
| Global p for IPV × survey period interaction | **<0.001** | **<0.001** | **0.001** |

**Note:** aPR, adjusted prevalence ratio; CI, confidence interval; IPV, intimate partner violence. The primary Model 2 adjusted for age group, educational level, union status, current work status, number of children living in the household, area of residence, natural region, wealth quintile, mother tongue, partner alcohol consumption, partner controlling behaviors, and father beating mother. The Model 1 sensitivity analysis excluded partner alcohol consumption, partner controlling behaviors, and father beating mother and adjusted only for the primary sociodemographic covariates. The complete-case Model 2 analysis was restricted to women with observed information for all Model 2 covariates. All models included an IPV × survey period interaction term and accounted for the ENDES complex survey design and health-module sampling weight. Global interaction p-values were obtained using design-adjusted Wald tests. The ratio of aPRs compares the magnitude of the IPV–depressive symptoms association in 2020–2021 with that in 2015–2019; values below 1 indicate a weaker relative association during 2020–2021.

**Source:** ENDES 2015–2024.
