# Supplementary material for: Intimate partner violence and depressive symptoms among ever-partnered Peruvian women: a repeated cross-sectional analysis of national surveys, 2015–2024
Source: Front Psychol. 2026 Oct 14;17:1939992. doi: 10.3389/fpsyg.2026.1939992 (PMC13713516; doi:10.3389/fpsyg.2026.1939992)
Supplement: Supplementary file 2 [file Data_Sheet_1.zip › Supplementary Material/STROBE Checklist.docx]

**STROBE Statement—Checklist of items that should be included in reports of cross-sectional studies**

**Manuscript: Intimate Partner Violence and Depressive Symptoms Among Ever-Partnered Peruvian Women: A Repeated Cross-Sectional Analysis of National Surveys, 2015–2024**

| **Section** | **Item** | **STROBE recommendation** | **Where reported in manuscript** | **Completion note** |
| --- | --- | --- | --- | --- |
| Title and abstract | 1a | Indicate the study’s design with a commonly used term in the title or the abstract. | Title and Abstract, pp. 1–2 | Repeated cross-sectional design stated in title and Methods of abstract. |
| Title and abstract | 1b | Provide in the abstract an informative and balanced summary of what was done and what was found. | Abstract, pp. 1–2 | Background, Methods, Results, and Conclusion reported with main estimates and non-causal interpretation. |
| Introduction | 2 | Explain the scientific background and rationale for the investigation being reported. | Introduction, pp. 2–3 | Global and Peruvian evidence, prior ENDES studies, and rationale for decade-long assessment are presented. |
| Introduction | 3 | State specific objectives, including any prespecified hypotheses. | Introduction, p. 3 | Objectives include trends in depressive symptoms, association with IPV, and temporal variation in the association. |
| Methods | 4 | Present key elements of study design early in the paper. | Methods 2.1, pp. 3–4 | Repeated cross-sectional secondary analysis of annual ENDES 2015–2024 surveys. |
| Methods | 5 | Describe the setting, locations, and relevant dates, including periods of recruitment, exposure, follow-up, and data collection. | Methods 2.1 and 2.4, pp. 4, 6–7 | National ENDES setting, survey years 2015–2024, and analytical periods are described. |
| Methods | 6a | Give eligibility criteria, and the sources and methods of selection of participants. | Methods 2.2, p. 4 | Eligibility, ever-partnered definition, module linkage, PHQ-9/IPV completeness, and weighting eligibility are reported. |
| Methods | 6b | For matched studies, give matching criteria and numbers of exposed and unexposed. | Not applicable | No matching was used. |
| Methods | 7 | Clearly define all outcomes, exposures, predictors, potential confounders, and effect modifiers. Give diagnostic criteria, if applicable. | Methods 2.4, pp. 5–7; Supplementary Tables S2–S3 | Outcome, IPV measures, period, covariates, and interaction term are operationalized. |
| Methods | 8 | For each variable of interest, give sources of data and details of methods of assessment. Describe comparability of assessment methods if there is more than one group. | Methods 2.3–2.4, pp. 5–7; Supplementary Tables S2–S3 | ENDES modules, PHQ-9 items, IPV source variables, recoding rules, and harmonization across years are documented. |
| Methods | 9 | Describe any efforts to address potential sources of bias. | Methods 2.3–2.5, pp. 5–10; Discussion limitations, pp. 15–16 | Privacy requirements, subpopulation survey estimation, sensitivity analyses, missing-data handling, and limitations are described. |
| Methods | 10 | Explain how the study size was arrived at. | Methods 2.2, p. 4; Supplementary Table S1 | Census of all eligible records across ten ENDES rounds after module linkage and analytic exclusions; sequential counts reported. |
| Methods | 11 | Explain how quantitative variables were handled in the analyses. If applicable, describe which groupings were chosen and why. | Methods 2.4, p. 7 | Age, education, children, wealth, and survey period categories are specified; survey year is also analyzed continuously and categorically. |
| Methods | 12a | Describe all statistical methods, including those used to control for confounding. | Methods 2.5, pp. 8–10 | Survey-weighted descriptive analyses and modified Poisson regression with Model 1 and Model 2 covariate sets are described. |
| Methods | 12b | Describe any methods used to examine subgroups and interactions. | Methods 2.5, p. 9 | IPV × survey-period interaction and pairwise contrasts are described. |
| Methods | 12c | Explain how missing data were addressed. | Methods 2.4, pp. 7–8; Supplementary Tables S1, S8 | Missing/not-reported categories, complete-case sensitivity analysis, and rationale for not using multiple imputation are reported. |
| Methods | 12d | If applicable, describe analytical methods taking account of sampling strategy. | Methods 2.5, pp. 8–9 | Health-module weights, strata, PSUs, pooled-year identifiers, and subpopulation/domain estimation are specified. |
| Methods | 12e | Describe any sensitivity analyses. | Methods 2.5, pp. 8–9; Supplementary Tables S7–S8 | Categorical-year analysis, exclusion of controlling behaviors, and complete-case analyses are reported. |
| Results | 13a | Report numbers of individuals at each stage of study (e.g., potentially eligible, examined for eligibility, confirmed eligible, included in the study, analyzed). | Results 3.1, p. 10; Supplementary Table S1 | Sequential sample construction from 372,972 women’s-file records to 101,145 weighted analytic observations is reported. |
| Results | 13b | Give reasons for non-participation at each stage. | Methods 2.2 and Results 3.1, pp. 4, 10; Supplementary Table S1 | Module-specific sampling/eligibility, not ever-partnered, incomplete PHQ-9/IPV, and non-positive/missing weight are documented. |
| Results | 13c | Consider use of a flow diagram. | Supplementary Table S1 | A detailed year-specific sequential selection table is provided; a flow diagram was not used. |
| Results | 14a | Give characteristics of study participants and information on exposures and potential confounders. | Results 3.1, p. 10; Table 1, pp. 22–23 | Sociodemographic, partner-related, IPV, and depressive-symptom characteristics are reported. |
| Results | 14b | Indicate number of participants with missing data for each variable of interest. | Methods 2.4, pp. 7–8; Supplementary Table S1 Panel B | Year-specific and overall missingness for selected covariates is reported. |
| Results | 14c | Summarize follow-up time, if appropriate. | Not applicable | Repeated cross-sectional study; no participant follow-up. |
| Results | 15 | Report numbers of outcome events or summary measures. | Results 3.1–3.2, p. 10; Table 1; Supplementary Table S5 | Overall and year-specific weighted prevalence of depressive symptoms is reported. |
| Results | 16a | Give unadjusted estimates and, if applicable, confounder-adjusted estimates and their precision. Make clear which confounders were adjusted for and why they were included. | Results 3.3–3.5, pp. 10–12; Tables 2–4; Supplementary Table S6 | Crude and adjusted PRs with 95% CIs and model covariates are reported. |
| Results | 16b | Report category boundaries when continuous variables were categorized. | Methods 2.4, p. 7; Table 1 | Category boundaries are shown for age, number of children, and survey periods; other categorical variables are explicitly defined. |
| Results | 16c | If relevant, consider translating estimates of relative risk into absolute risk for a meaningful time period. | Not applicable | The study estimates prevalence ratios in cross-sectional data rather than incident risks over a follow-up period. |
| Results | 17 | Report other analyses done—e.g., analyses of subgroups and interactions, and sensitivity analyses. | Results 3.4–3.5, pp. 11–12; Supplementary Tables S7–S8 | Interaction, pairwise contrasts, and sensitivity analyses are reported. |
| Discussion | 18 | Summarize key results with reference to study objectives. | Discussion, p. 12 | Opening Discussion paragraph summarizes the main association, subtype findings, trends, and temporal heterogeneity. |
| Discussion | 19 | Discuss limitations of the study, taking into account sources of potential bias or imprecision. | Discussion, pp. 15–16 | Temporality, measurement, selection, privacy, weighting, missingness, overadjustment, survey-mode changes, modeling, and generalizability are discussed. |
| Discussion | 20 | Give a cautious overall interpretation of results considering objectives, limitations, multiplicity of analyses, results from similar studies, and other relevant evidence. | Discussion, pp. 12–16 | Findings are compared with prior studies and interpreted as associations/cross-sectional co-occurrence rather than causal effects. |
| Discussion | 21 | Discuss the generalisability (external validity) of the study results. | Discussion, pp. 14–16 | Generalisability is limited to ever-partnered women aged 15–49 years represented by ENDES. |
| Other information | 22 | Give the source of funding and the role of the funders for the present study and, if applicable, for the original study on which the present article is based. | Funding, p. 17 | The authors report no specific grant from public, commercial, or not-for-profit funding agencies. |
